# Embryo vitrification disturbs the pre-implantation DNA demethylation process and affects hepatic functions to threaten long-term metabolic health in mice

**DOI:** 10.1016/j.jare.2025.11.032

**Published:** 2025-11-17

**Authors:** Yue Ying, Yang Yang, Xilin Shen, Yuli Qian, Jianpeng Chen, Yanyun Ying, Hao Jin, Yazeed Allan, Yifeng Lin, Feixia Wang, Juan Liu, Junyan Zheng, Jiani Jin, Yifeng Liu, Qingyuan Sun, Hongqing Liang, Weimin Ci, Dan Zhang, Chuan Chen

**Affiliations:** a Institute of Medical Genetics and Development, Key Laboratory of Reproductive Genetics (Ministry of Education) and Department of Reproductive Endocrinology, Women’s Hospital, School of Medicine, Zhejiang University, Hangzhou 310006, China; b China National Center for Bioinformation, Beijing 100101, China; c Beijing Institute of Genomics, Chinese Academy of Sciences, Beijing 100101, China; d University of Chinese Academy of Sciences, Beijing 100049, China; e Department of Nutrition, Harvard T.H. Chan School of Public Health, Boston 02115, USA; f Center for Reproductive Medicine, The First Affiliated Hospital, School of Medicine, Zhejiang University, Hangzhou 310003, China; g Zhejiang Provincial Birth Defect Control and Prevention Research Center, Hangzhou 310006, China; h Guangzhou Key Laboratory of Metabolic Diseases and Reproductive Health, Guangdong-Hong Kong Metabolism & Reproduction Joint Laboratory, Reproductive Medicine Center, Guangdong Second Provincial General Hospital, Guangzhou 510317, China; i Division of Human Reproduction and Developmental Genetics, Key Laboratory of Reproductive Genetics (Ministry of Education), Women’s Hospital, School of Medicine, Zhejiang University, Hangzhou 310006, China; j Department of Urology, Chinese PLA General Hospital, Beijing 100039, China

**Keywords:** Embryo vitrification, Tet2, DNA methylation, Metabolic disturbances, Lipid metabolism-associated genes

## Abstract

**Introduction:**

Embryo vitrification has been linked to an increased incidence of large-for-gestational-age offspring. Investigating how epigenetic regulation transmits vitrification-induced abnormalities to postnatal stages could provide valuable insights for the control and prevention of metabolic disorders in offspring derived from vitrified-thawed embryos, yet the underlying mechanisms remain poorly understood.

**Objectives:**

This study aimed to investigate vitrification-induced aberrations in gene expression and DNA methylation remodeling during pre-implantation stages, and to explore how these abnormalities affect the postnatal metabolic health of the offspring.

**Methods:**

8-cell mouse embryos were vitrified and thawed, and transcriptome profiling was conducted using SMART-seq2 with 16-cell embryos, morulae and blastocysts. DNA methylome profiling of blastocysts using low-input bisulfite sequencing (LI-BS) was then performed. After embryo transfer, glucose tolerance tests (GTT) and insulin tolerance tests (ITT) were conducted on the offspring, and blood metabolite levels were measured by ELISA. Histological analyses, RNA-seq, and whole-genome bisulfite sequencing (WGBS) were then performed on hepatic tissues of the offspring.

**Results:**

Here, we found delayed progression of vitrified-thawed embryos, with downregulated expression of *Tet2*. Consistently, we observed genome-wide DNA hypermethylation, with the hypermethylated genes being enriched in metabolic processes. Our data further demonstrated that offsprings from vitrified-thawed embryos exhibited metabolic disturbances including insulin resistance, lipid deposition and mitochondrial dysfunction. Interestingly, genes associated with arachidonic acid metabolism remained dysregulated in the hepatic tissues of these offspring, although DNA methylation had been restored to the normal level in these terminally differentiated tissues.

**Conclusions:**

Overall, our study uncovers the negative impact of vitrification on DNA methylation remodeling during early embryo development, which may partially account for the elevated susceptibility to metabolic diseases in offspring. Our findings also suggest that the *Tet2* gene is a potential therapeutic target for improving the developmental competence of vitrified-thawed embryos.

## Highlights

• Embryo vitrification-thawing represses *Tet2 and causes* pre-implantation DNA hypermethylation.
• OPA1 restores *Tet2 expression and the global* DNA methylation level in vitrified-thawed embryos.
• Hypermethylated genes in vitrified-thawed embryos are associated with metabolic processes.
• Offspring derived from vitrified-thawed embryos exhibit metabolic disturbances.
• Lipid metabolism genes are downregulated in livers of offspring from vitrified-thawed embryos.

## Graphical abstract

**Figure:**
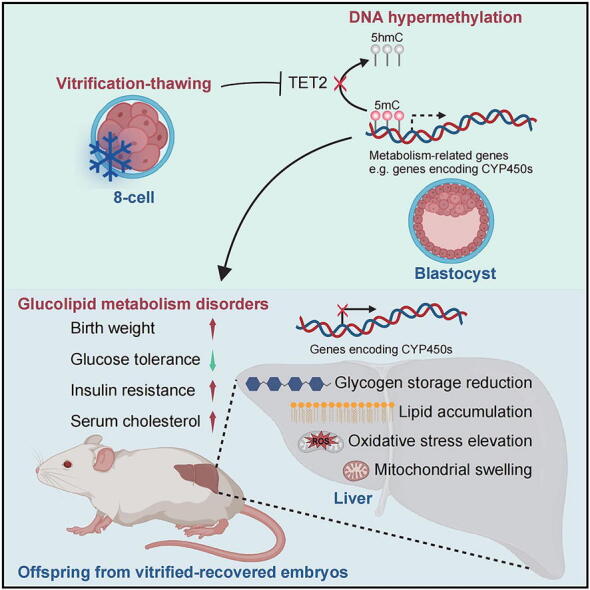

## Introduction

With the widespread clinical application of assisted reproductive technology (ART) to treat infertility, cryopreservation techniques are routinely performed for long-term storage of oocytes and embryos, providing a reliable way of fertility preservation.[1] The vitrification technique ensures embryos to be flash frozen and transformed into a glass-like condition following treatment with high-concentration cryoprotectants to avoid ice crystal formation, and vitrified embryos are recovered in thawing solution before the embryo transfer.[2,3] In recent years, vitrification has replaced slow-freezing as the commonly used method for embryo cryopreservation owing to more efficient procedures as well as higher rates of embryo survival, implantation and pregnancy.[4,5] However, the threat of frozen embryo transfer (FET) to the health status of offspring has been widely concerned, as FET leads to increased risk of large-for-gestational-age (LGA) birth weight, macrosomia and type 1 diabetes compared to fresh embryo transfer.[[6], [7], [8], [9], [10], [11]] Research in mouse models also demonstrated that embryo vitrification could lead to altered metabolic phenotypes in offspring.[12] However, the underlying mechanisms by which manipulation of preimplantation embryos exerts adverse effects on long-term metabolic health remain poorly understood.

During early mammalian development, dramatic epigenetic reprogramming occurs to ensure the conversion from terminally differentiated gametes to a totipotent zygote.[[13], [14], [15]] As one of the major epigenetic remodeling processes, genome-wide DNA demethylation initiates shortly after fertilization and continues until the final pre-implantation stage, thereby resulting in minimal methylation levels at the blastocyst stage.[[16], [17], [18], [19], [20], [21]] TET proteins have been proven to mediate this process through catalyzing the conversion of 5-methylcytosine (5mC) of DNA to 5-hydroxymethylcytosine.[22,23] This remodeling equalizes the distinct parental methylomes and reverts cellular memory towards an undifferentiated state, which is required for the emergence of pluripotent cells during early development.[15] Since the global DNA methylation is quickly reestablished after implantation,[24,25] an incomplete elimination of the methylome at the pre-implantation stages may impede the epigenetic reprogramming as well as appropriate lineage specification, leading to developmental defects in subsequent stages.[26] Indeed, depletion of *Tet* genes in mouse embryos caused severe developmental arrest, and insufficient TET-mediated DNA demethylation in early embryos resulted in metabolic phenotypes after birth.[27,28].

Given that the epigenetic remodeling is crucial for subsequent development and highly susceptible to environmental interference, it has been widely accepted that adverse exposures during the pre-implantation window threaten adult health through epigenetic dysregulation.[29,30] In this context, such epigenetic abnormalities emerging in early embryos are considered to serve as the developmental origin of adult diseases. Although several studies suggest that vitrification may be associated with aberrant epigenetic modifications in mouse embryos and fetuses,[[31], [32], [33], [34], [35]] whether these changes are associated with the increased risk of metabolic disturbances in offspring remain controversial.

Here, we focused on the gene expression alterations in mouse embryos following vitrification-thawing at the 8-cell stage, and noticed the downregulation of *Tet2*. Consistently, our immunofluorescence staining and bisulfite sequencing revealed an elevation in the global DNA methylation level in vitrified-thawed blastocysts, with the functions of hypermethylated genes being enriched in metabolic processes. We hypothesized that vitrification disrupts TET2-mediated DNA demethylation, leading to metabolic dysregulation. We then checked the status of glucose and lipid metabolism in offspring from vitrified-thawed embryos, and observed insulin resistance as well as accumulated lipid storage and mitochondrial dysfunctions in hepatic tissues. Finally, we demonstrated altered expression levels of metabolism-associated genes in hepatic tissues of offspring from vitrified-thawed embryos despite the normal methylation levels. Collectively, our findings suggest that embryo vitrification disturbs the DNA methylation reprogramming at pre-implantation stages, particularly affecting metabolism-associated genes. These epigenetic perturbations may serve as a developmental origin, contributing to the increased incidence of metabolic disorders in offspring derived from vitrified-thawed embryos.

## Material and Methods

### Mice

ICR mice were purchased from Zhejiang Vital River Laboratory Animal Technology (#201, Charles River Laboratories). All mice were housed under the specific pathogen-free condition in The Laboratory Animal Center of Zhejiang University, and kept on a 12-hour (h) light/dark cycle (light from 7:00 AM to 7:00 PM) at 21–25 °C and 40–70 % relative humidity with free access to food and water.

### Mouse embryo retrieval and *in vitro* culture

6- to 8-week-old virgin female ICR mice and 8- to 10-week-old male ICR mice were used to mate and obtain embryos. Female mice were superovulated by intraperitoneal injection of 5 IU Pregnant Mare’s Serum Gonadotropin (PMSG; Hangzhou Animal Medicine Factory) followed by intraperitoneal injection of 5 IU human Chorionic Gonadotropin (hCG, Hangzhou Animal Medicine Factory) 48 h later. Each primed female was mated with a single male breeder, and the next morning was marked as 0.5 day post coitum (dpc) if the vaginal plug was detected. To collected embryos at 8-cell, 16-cell, morula or blastocyst stage, female mice with vaginal plugs were sacrificed at 2.5, 2.75, 3 or 3.5 dpc respectively, and embryos were flushed from the oviducts with HEPES-supplemented M2 Medium (#M7157, Sigma-Aldrich). 16-cell embryos, morulae and blastocysts were directly collected for the *in vivo group*, whereas 8-cell embryos were subjected to *in vitro* culture within KSOM (ARK Resource) droplets covered by Mineral oil (#M8410, Sigma-Aldrich) in a humidified incubator at 37 °C under 5 % CO_2_ for the *in vitro group*, or vitrification-thawing before *in vitro* culture for the *vitrified group*. 8-cell embryos were cultured 0.25, 0.5 or 1 day to obtain 16-cell, morula or blastocyst embryos, and only embryos morphologically corresponding with designated developmental stages were collected for subsequent experiments. For statistical analysis of embryo development rates, the initial numbers of embryos among all experimental groups were no less than thirty, which meets the sample size requirement (see Sheet 1 of Table S1 for details).

### Mouse embryo vitrification and thawing

Vitrification and thawing of the 8-cell embryos were performed using CRYOTOP Vitrification Kit and Thawing Kit (#VT101 and #VT102, Kitazato), respectively, according to the manufacturer′s instructions. Briefly, the embryos were placed into the equilibration solution (ES) droplet for 3–5 min (min) and the vitrification solution (VS) droplet for 45 s before they were transferred to the tip of a cryostraw (Kitazato), and the embryo-containing cryostraw was immediately submerged into the liquid nitrogen. For embryo recovery, the vitrified embryos were promptly placed into the thawing solution (TS) droplet at 37 °C for 1 min before they were transferred to the diluent solution (DS) droplet and incubated for 3 min. The embryos were then placed into the washing solution1 (WS1) for 5 min followed by three washes in WS2. Finally, the thawed embryos were transferred into KSOM and incubated at 37 °C under 5 % CO_2_ to recover for 2 h before these 8-cell embryos were cultured sequentially for 0.25, 0.5 or 1 day to obtain 16-cell, morula or blastocyst embryos. For loperamide hydrochloride (OPA1) treatment, thawed embryos were cultured in KSOM supplemented with 500 nM OPA1 (#B33838, Shanghai Yuanye Bio-Technology; dissolved in 0.05 % DMSO) until harvest, and embryos cultured in KSOM supplemented with 0.05 % DMSO served as the control.

### Mouse embryo transfer

For induction of pseudopregnancy, 8-week-old female ICR mice were mated with 10-week-old surgically vasectomized male ICR mice, and females with copulatory plugs the next morning were regarded as pseudopregnant recipients at 0.5 dpc. At 2.5 dpc, these recipients were anesthetized by intraperitoneal injection of 1.25 % avertin (0.2 mL per 10 g body weight; #T48402, Sigma-Aldrich), and blastocysts from each group were transferred into the uterine lumen with each uterine horn receiving 7 embryos, for a total of 14 embryos transferred to each recipient under the stereomicroscope through the embryo manipulation pipette after the uterine wall was punctured by a narrow needle. Then the skin was sutured, and the recipients were placed on a heating pad for postoperative recovery before they were moved back to the cage. The numbers of transferred embryos, recipients, newborn pups and adult offspring as well as sex information of the offspring for each group are included in Sheet 2 of Table S1.

### Immunofluorescence (IF) staining of embryos

Embryos were fixed in 4 % PFA for 30 min at room temperature and washed 3 times with 1 % bovine serum albumin (BSA; #V900933, Sigma-Aldrich) in PBS. The embryos were then permeabilized at room temperature for 20 min in 0.5 % Triton X-100 (#T9284, Sigma-Aldrich) for TET2 staining or 2 M HCl, 0.1 % Triton X-100 for 5mC/5hmC staining, and blocked with 1 % BSA in PBS for 1 h at room temperature. Thereafter, the embryos were incubated with primary antibodies at 4 °C overnight before they were washed three times with 1 % BSA in PBS and incubated with secondary antibodies for 1 h at room temperature followed by DAPI (#ab228549, Abcam) staining. The primary antibodies were used as follows: Rabbit monoclonal anti-TET2 (1:400; #36449, RRID: AB_2799102, clone D6C7K, Cell Signaling), Rabbit monoclonal anti-5mC (1:800; #28692, RRID: AB_2798962, clone D3S2Z, Cell Signaling), Mouse monoclonal anti-5hmC (1:200; #51660, RRID: AB_2799398, clone HMC31, Cell Signaling). The secondary antibodies were used as follows: Goat anti-Mouse IgG, Alexa Fluor 488 (1:500; #A-11001, RRID: AB_2534069, Invitrogen), Dylight 594, Goat Anti-Rabbit IgG (1:500; A23420). Finally, the embryos were observed and imaged under a confocal spinning-disk microscope (Olympus), and image analyses were conducted using Imaris software with 10–13 biological replicates for each group.

### Metabolic phenotype assessment

Body composition represented by the content of fat, lean tissues and free water in 5-week-old conscious mice was assessed after 4-h fasting using the low-field nuclear magnetic resonance (NMR) small animal body composition analyzer (Suzhou Niumag Analytical Instrument Corporation), and 22–27 offspring were measured for each sex in each group. Glucose metabolism was measured for 4-, 8-, 12- or 16-week-old mice through the glucose tolerance test (GTT), which was performed at 9 a.m. after 16-h fasting, and D-Glucose (#Y0001745, Sigma-Aldrich) was administrated via intraperitoneal injection (2 g per 1 kg body weight). The blood glucose levels were then measured using a blood glucose monitor (Roche Diagnostics) at 0, 30, 60, 90 and 120 min post injection. For insulin tolerance test (ITT), 5-, 9, 13- or 17-week-old mice were intraperitoneally injected with Recombinant Insulin (Wanbang Biopharmaceuticals; 0.8 IU per 1 kg body weight), and the blood glucose levels were measured at 0, 30, 60, 90 and 120 min post injection. For the GTT and ITT, 21–30 offspring were measured for each sex in each group, and the area under the curve (AUC) of the glucose levels was calculated according to the trapezoidal rule.[36].

To assess the level of lipid/glucose-related metabolites and insulin in serum, blood samples were collected from the *retro*-orbital sinus puncture of the 10- or 18-week old anesthetized mice after 4-h fasting, and the serum was then separated from the blood and stored at −80 °C for further analyses. The levels of representative metabolites including GSP, cholesterol, triglyceride and LDL-C were determined by the biochemical analyzer (Toshiba), and the serum insulin level was assessed using Mouse Insulin ELISA Kit (#KE10089, Proteintech). The HOMA-IR index was calculated using the formula: HOMA-IR = (fasting insulin [μU/mL] × fasting glucose [mmol/L]) / 22.5. For each metabolite assessment, 12–16 offspring were measured for each sex in each group.

### Tissue histological analysis

Hepatic tissues were collected from 10-week-old female mice after 4-h fasting and fixed overnight with 10 % neutral buffered formalin for paraffin embedding or 4 % paraformaldehyde (PFA) for cryosections. After deparaffinization and rehydration, PAS staining was performed on the paraffin sections using 0.5 % Periodic Acid Solution (#100482, Sigma-Aldrich) for polysaccharide oxidization and Schiff’s Reagent (#3952016, Sigma-Aldrich) for visualization of the oxidized compound. The stains were then washed with lukewarm water and counterstained with Hematoxylin Solution according to Mayer (1.044 g/mL; #51275, Sigma-Aldrich). For ORO staining, the PFA-fixed tissues were dehydrated in 30 % sucrose solution and embedded in O.C.T. Compound (#4583, Sakura Finetek) before they were frozen at −80 °C. 10-μm cryosections were made and stained with Oil Red O Solution (0.5 % in isopropanol; #O1391, Sigma-Aldrich) and Mayer’s Hematoxylin Solution. All sections were observed and imaged under a light microscope (Olympus), and image analyses were conducted using ImageJ software with 4 biological replicates for each group. The mean gray values and the integrated density gray values were measured to quantify the glycogen storage and the lipid droplets, respectively.

### Microstructure analysis by transmission electron microscopy (TEM)

Hepatic tissues collected from 10-week-old female mice were fixed with 2.5 % glutaraldehyde (#30092436, Sinopharm Chemical Reagent) at room temperature for 2 h and 4 °C overnight. After three 10-min washes in PBS, the tissues were further fixed with 1 % osmic acid (#18456, Ted Pella) at room temperature for 1 h followed by three 10-min washes in PBS. The tissues were then sequentially treated with 2 % uranyl acetate (#02624-AB, SPI Supplies) for 30-min fixation and staining, 50 %, 70 %, 90 % and 100 % ethanol for 10-min dehydration each, 100 % acetone (#10000418, Sinopharm Chemical Reagent) for 15-min washing twice, 50 % acetone diluted with the embedding agent, PON 812 Epoxy Resin Monomer (#02659R-AB, SPI Supplies) for 2-h infiltration and 25 % acetone diluted with the embedding agent overnight before they were embedded in the embedding agent at 37 °C for 12 h and 65 °C for 48 h to allow polymerization. The plastic blocks were then cut into 60–80 nm sections using an ultramicrotome (Leica). Sections were counterstained with 2 % uranyl acetate and 2.6 % lead citrate (#180705-C5382, Electron Microscopy Sciences) for 8 min respectively and allowed to air-dry overnight. The ultrastructure of the hepatic tissues was observed and imaged under a cryogenic electron microscope (FEI), and the minor-to-major axis ratios of mitochondria were measured using ImageJ software with 12–14 biological replicates for each group.

### Oxidative stress-related substance level detection

Fresh hepatic tissues were dissected from 10-week-old female mice and washed thoroughly with saline to remove blood clots and red blood cells, and homogenization was completed in ice-cold phosphate-buffered saline (PBS; 9 mL per 1 g tissues) before the centrifugation at 5,000 rpm for 15 min. The supernatant was then subjected to the spectrophotometric analysis using ROS ELISA Kit (#MM-43700 M2, Jiangsu Meimian Industrial), Malondialdehyde (MDA) Assay Kit (#G4300, Servicebio), Total Superoxide Dismutase (T-SOD) Activity Assay Kit (#G4306, Servicebio), Glutathione Peroxidase (GSH-PX) Assay Kit (#A005, Nanjing Jianchen) and Catalase (#G4307, CAT) Activity Assay Kit (Servicebio) according to the manufacturers' instructions. For each assay, 6 biological replicates were measured for each group.

### Embryo mRNA-seq library preparation by SMART-seq2

The mRNA-seq libraries for early embryos were prepared according to the published protocol[37] with minor modifications, and five embryos were pooled per replicate, with 4–8 replicates performed per group. Briefly, embryos were washed three times with 0.1 % BSA in PBS before the embryos for each library were transferred to 4 μL cell lysis buffer composed of 0.5 % Triton X-100, 2 U/μL RiboLock RNase Inhibitor (#EO0381, Thermo Scientific), 2.5 μM oligo-dT_30_VN primer (5′-AAGCAGTGGTATCAACGCAGAGTACT_30_VN-3′) and 2.5 μM each dNTP Mix (#R0192, Fermentas), and the tubes were vortexed and incubated at 72 °C for 3 min. The reverse transcription mix was then added to the sample to prepare a 10-μL reaction containing the lysate from the previous step, 10 U/μL SuperScript II Reverse Transcriptase (#18064–014, Invitrogen), 1 × Superscript II First Strand Buffer, 2 U/μL RNase Inhibitor, 5 mM DTT, 1 M Betaine (#61962, Sigma-Aldrich), 6 mM MgCl_2_ and 1 μM template-switching oligo (TSO; 5′-AAGCAGTGGTATCAACGCAGAGTACATrGrG + G-3′) for each library, and the reaction was mixed and incubated at 42 °C for 90 min, 10 cycles of 50 °C for 2 min and 42 °C for 2 min, and 70 °C for 15 min. PCR preamplification was then performed after the addition of the ISPCR primer (5′-AAGCAGTGGTATCAACGCAGAGT-3′) and KAPA HiFi HotStart ReadyMix PCR Kit (#KK2602, KAPA Biosystems), and the amplified DNA was purified twice with 0.8 × AMPure XP Beads (#A63881, Beckman Coulter). The libraries were then prepared using TruePrep DNA Library Prep Kit V2 for Illumina (#TD503, Vazyme) along with TruePrep Index Kit V2 for Illumina (#TD202, Vazyme) following the manufacturer′s instructions. Finally, the libraries were sequenced on the Illumina NovaSeq 6000 (Novogene) producing 150-bp pair-end reads.

### Tissue mRNA-seq library preparation

Hepatic tissues were collected from 10-week-old female mice. The mRNA-seq libraries for hepatic tissues were prepared using TruSeq RNA Library Prep Kit v2 (#RS-122–2001, Illumina) following the manufacturer′s instructions, and 3 biological replicates were performed per group. Briefly, total RNA from tissues were extracted and 1 μg RNA per library was subjected to mRNA isolation by oligo-dT beads followed by RNA fragmentation with the fragmentation buffer. First and second strand synthesis, end repair and phosphorylation, A-tailing and index adapter ligation were sequentially performed. The cDNA libraries were then subjected to size selection for 200 to 300 base-pair (bp) fragments using 2 % Certified Low Range Ultra Agarose (#1613106, Bio-Rad), and a 15-cycle PCR amplification was conducted with Phusion High-Fidelity DNA Polymerase (#M0530S, New England Biolabs). Finally, 150-bp pair-end sequencing was performed on the Illumina NovaSeq 6000 platform (Shanghai BIOZERON Biotechnology).

### Embryo low-input bisulfite sequencing (LI-BS) library preparation

The DNA methylation libraries of blastocysts were prepared according to the published single-cell bisulfite sequencing method,[38] and each replicate contained a single embryo, with 11–13 replicates per group. Briefly, zonae pellucidae were dissolved and removed with EmbryoMax Acidic Tyrode’s Solution (#MR-004, Sigma-Aldrich), and the embryos were washed three times with 0.1 % BSA in PBS. Then, the embryos were transferred to Buffer RLT Plus (#1053393, Qiagen) and lysed on ice for 2 h. The released DNA was then subjected to bisulfite conversion and purification with EZ DNA Methylation-Direct Kit (#D5020, Zymo Research), and the purified DNA was pre-amplified for 5 cycles with the addition of Preamp Oligo (5′-TCGTCGGCAGCGTCAGATGTGTATAAGAGACAGNNNNNN-3′), TIANSeq Klenow (3′-5′ exo-; #NG202-02, Tiangen Biotech) and dNTP Mix to increase the starting materials and complete the first strand labeling. Thereafter, excess primers were degraded using Exonuclease I (#M0293L, New England Biolabs), and the products were purified using AMPure XP Beads before the second-strand labeling was completed with Adapter 2 Oligo (5′-GTCTCGTGGGCTCGGAGATGTGTATAAGAGACAGNNNNNN-3′), the Klenow enzyme and dNTP Mix. The double-tagged products were purified by XP Beads and then subjected to library amplification for 14 to 17 cycles with KAPA HiFi HotStart ReadyMix PCR Kit and TruePrep Index Kit V2 for Illumina, and the amplified products were purified with XP Beads. Finally, the libraries with a peak fragment size of approximately 500 bp were selected for 150-bp pair-end sequencing on the Illumina NovaSeq 6000 (Novogene).

### Tissue whole-genome bisulfite sequencing (WGBS) library preparation

Hepatic tissues were collected from 10-week-old female mice for DNA methylation library preparation, and 3 biological replicates were performed per group. Briefly, genomic DNA was extracted, and 200 ng DNA was used for each reaction with the addition of unmethylated Lambda DNA (#D152A, Promega) at a mass ratio of 3:1000. DNA fragmentation was performed using a sonicator (Covaris) at 4 °C to produce 400-bp fragments, and the fragmented DNA was then subjected to end-repair and A-tailing using NEBNext Ultra II DNA Library Prep Kit for Illumina (#E7645L, New England Biolabs) following the manufacturer′s instructions. The products were purified with 0.9 × AMPure XP Beads and methylated adapters were ligated to the DNA. After the DNA purification with 0.9 × XP Beads, bisulfite conversion and DNA recovery were performed using EZ DNA Methylation-Gold Kit (#D5005, Zymo Research), followed by PCR amplification with KAPA HiFi HotStart Uracil + Kit (#KK2801, KAPA Biosystems). Finally, the libraries were subjected to 150-bp pair-end sequencing on the Illumina NovaSeq 6000 (Novogene).

### mRNA-seq data processing and analyses

Sequencing reads were trimmed by Trim Galore (v0.6.6; Babraham Bioinformatics) to remove adapter sequences with the parameters: −phred33 −illumina −clip_R1 9 −clip_R2 9 −paired, and the trimmed reads were mapped to mouse genome version mm10 using the unique alignment mode of TopHat2[39] (v2.1.1). The BAM files containing mapped reads were downsampled using the DownsampleSam function of Picard Tools (Broad Institute) to normalize the sequencing depth before calculating the gene counts. The read mapped to each gene was then counted using featureCounts[40] (v2.0.3) with the following parameters: −primary −M −p −B −C −t exon −g gene_id, and the expression level was quantified as transcripts per million (TPM) in R. Correlation between each replicate was calculated and principal component analysis (PCA) was performed with gene TPM in R. Pseudotime analysis of the embryo mRNA-seq was carried out using the R package Monocle[41] following the recommended analysis protocol, and the expression matrix containing unnormalized gene counts calculated by featureCounts served as the input data. To allow direct comparison between our dataset and the published data, we used the R package DESeq2 to remove the batch effect and extract the normalized gene counts. Differentially expressed genes (DEGs) were identified using the normalization method provided by the edgeR package of R Bioconductor[42], with the cutoff of P value < 0.05. Functional enrichment analysis of gene sets was performed using the R package clusterProfiler (v4.10.1), and significant gene ontology (GO) clusters with P value < 0.05 were summarized to representative terms. Gene Set Enrichment Analysis (GSEA) algorithm[43] was used to visualize the concordant expression differences of a specific set of genes between two groups.

### LI-BS and WGBS data processing and analyses

Sequencing reads were trimmed for adaptor sequences using Trim Galore and quality control was performed with FastQC (v0.11.9; Babraham Bioinformatics). The mouse mm10 reference genome and the lambda DNA reference were prepared using the bismark_genome_preparation command of Bismark[44] (v0.16.1). The reads were then mapped to the genomes with Bismark, and the −–non_directional parameter was used when mapping LI-BS data. To evaluate the bisulfite conversion efficiency, non-CpG methylation levels were quantified using MethylExtract (v1.9.1) as previously described[38] for blastocyst samples, and samples with a conversion rate higher than 95 % were considered acceptable for further analysis; the conversion rate of cytosines in lambda DNA was calculated for liver samples, and samples with a conversion rate higher than 98 % were kept. Duplicate sequences were identified and removed using the deduplicate_bismark command of Bismark when generating the BAM files. The aligned sequences were then sorted by their header and left-to-right positions using the sort command of samtools[45] (v1.17). Copy number variations (CNVs) in the BAM files were calculated using ichorCNA[46] (v0.2.0), and samples with significant CNVs were excluded. In this study, the DNA methylation level of a designated region for each sample refers to the methylation rate of CpG sites within the region. This rate was calculated as the number of methylated CpG sites within a specific region divided by the total number of CpG sites identified in this region, and each region should be covered at least 3 times. This approach ensures robust methylation statistics that accurately reflect biological variations, enabling a more precise interpretation of the methylation landscape across diverse samples. To identify differentially methylated regions (DMRs), bismark_methylation_extractor command of Bismark was used to extract methylation information at each cytosine site, including the number of methylated reads, the number of unmethylated reads, and the methylation rate. With this information, candidate DMRs were identified using the R package, DSS[47] (v0.36.1), and regions with P value < 0.05, absolute methylation differences > 0.2 and more than three CpG sites were considered as significant DMRs. The R package ChIPseeker (v1.32.1) was used to annotate DMRs to genomic features including promoters, untranslated region (UTR), exons, introns and intergenic regions.

### Statistical analysis

For all statistical analyses, normality was assessed by the Shapiro-Wilk normality test using the shapiro.test function in RStudio (R version 4.2.0). When variables presented normal distribution, the Student’s *t*-test was performed for the comparison between two groups, and one-way analysis of variance (ANOVA) was applied for multiple group comparisons. Data that were not normally distributed were analyzed using the Mann–Whitney test or the Kruskal–Wallis test.

For bioinformatic data, the analyses and the plotting were performed in R. The two-sided paired Student’s *t*-test, one-way ANOVA, Mann–Whitney test and Kruskal–Wallis test were performed using the t.test, aov, wilcox.test and kruskal.test functions, respectively. The mean values of replicates were utilized for graphical representation unless otherwise stated.

For animal experiments, statistical analyses were performed in GraphPad Prism 9. Quantitative analyses of image information were conducted using ImageJ and Imaris software. Data were shown as the mean ± the standard error of the mean (SEM).

The standard designation of *P* values was applied throughout the figures (n.s. indicated not significant or *P* ≥ 0.05; *P < 0.05, **P < 0.01, ***P < 0.001, and ****P < 0.0001) unless otherwise stated.

## Results

### The development progress of mouse embryos is delayed upon vitrification-thawing

**Fig. 1:**
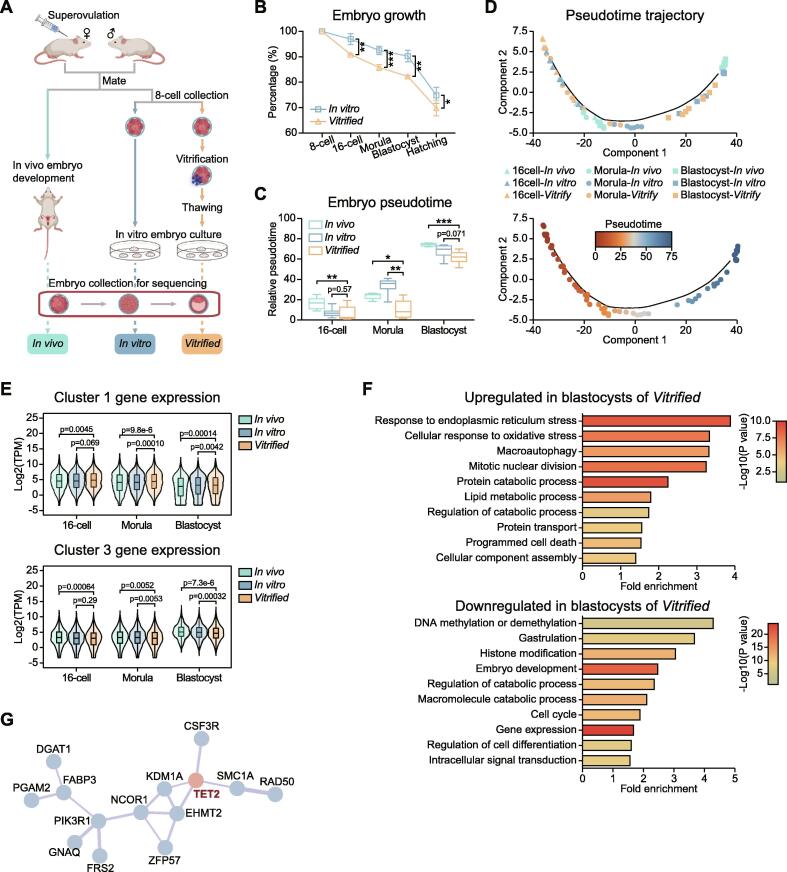
**The development progress of mouse embryos is delayed upon vitrification-thawing. (A)** Strategy for obtaining pre-implantation embryos of each group. 16-cell embryos, morulae and blastocysts were directly collected from ICR female mice following mating at 2.75, 3 or 3.5 day post coitum (dpc) respectively for the *in vivo group*, whereas 8-cell embryos were collected and subjected to *in vitro* culture for the *in vitro group* or vitrification-thawing before vitro culture for the *vitrified group*. Only embryos displaying morphological features consistent with the designated developmental stage were harvested for subsequent analyses. Some elements in this figure were adapted from BioRender.com. **(B)** Line charts displaying the embryo development rate at each stage. Percentages were calculated based on the number of embryos reaching each stage at the beginning of the next stage. Five biological replicates were tested for each group, with each replicate consisting of at least 45 initial embryos (see Sheet 1 of Table S1 for details). **(C)** Box plots showing the pseudotime values in embryo mRNA-seq replicates analyzed by Monocle (*n* = 8 for each sample). **(D)** Trajectory plot showing the developmental progression of embryo mRNA-seq replicates analyzed by Monocle. Replicates are ordered along the x-axis in pseudotime based on the expression levels of top 3,000 genes showing significant changes during stage transitions. The legends represent the types of replicates (upper panel) and the pseudotime (lower panel), respectively. **(E)** Violin plots with embedded box plots showing the expression levels of Cluster 1 genes (upper panel; *n* = 1694) and Cluster 3 genes (lower panel; *n* = 1157) in embryos at each stage. These genes were identified by k-means clustering (see Fig. S1D). **(F)** Horizontal bar charts displaying the top enriched functional categories for upregulated genes (upper panel) and downregulated genes (lower panel) identified in the *vitrified group* at the blastocyst stage. Fold enrichment and P values were calculated by clusterProfiler. **(G)** Network diagram provided by the STRING database illustrating the protein–protein interactions. These proteins are encoded by genes downregulated in both morulae and blastocysts of the *vitrified group*, and proteins lacking any interaction with each other were excluded.

To clarify the impact of vitrification on mouse early embryo development and exclude the effects caused by *in vitro* culture, we designed the *in vivo group* and the *in vitro group* as the controls for the *vitrified group* (Fig. 1A). Considering that human embryo vitrification is commonly conducted at cleavage stages around the 8-cell stage during the medically assisted reproduction,[48,49] we collected 8-cell mouse embryos for the vitrification in this study. Most of the vitrified embryos were successfully recovered, and the developmental ability at subsequent pre-implantation stages was consistently decreased in the *vitrified group* compared to the *in vitro group* (Fig. 1B and Sheet 1 of Table S1). To uncover the underlying defects caused by vitrification, we collected 16-cell embryos, morulae and blastocysts of each group and performed transcriptome sequencing using SMART-seq2.[37,50] We collected embryos exhibiting typical morphologies at each stage for sequencing library preparation, and validated a good reproducibility for the replicates (Fig. S1A, B). Notably, the vitrified-thawed embryos showed transcriptomic differences at the morula stage, as morulae of the *vitrified group* tended to share more similarities with 16-cell embryos of the control groups (Fig. S1C). The differences between the *vitrified group* and the *in vitro group* were reduced at the blastocyst stage, indicating that parts of the transcriptome defects caused by vitrification might be restored during subsequent development. Consistently, pseudotime analysis of the mRNA-seq data revealed that progression of the vitrified-thawed embryos was delayed, especially at the morula stage (Fig. 1C, D).

We next sought to investigate the detailed changes in transcriptomic dynamics of the *vitrified group*. To identify genes that contribute most to the transcriptomic transition during development, we selected top 3000 genes showing the most significant changes in expression throughout the developmental trajectory using Monocle's differential analysis toolkit,[41] and applied *k*-means clustering to these genes based on their expression patterns (Fig. S1D and Sheet 1 of Table S2). Notably, genes of cluster 1 and cluster 3, the two clusters containing the largest number of genes, were dysregulated in the *vitrified group* (Fig. 1E). Cluster 1 and cluster 3 genes were supposed to be downregulated and upregulated respectively after the 16-cell stage, and they exhibited higher or lower expression levels in morulae or blastocysts of the *vitrified group*, suggesting an inefficient transition of the gene expression profile in vitrified-thawed embryos. Therefore, the global transcriptomic transition across the pre-implantation stages is dysregulated in the *vitrified group*, indicating that the vitrification and thawing procedures may impede the developmental progression of mouse embryos, and the abnormal gene expression pattern observed in vitrified-thawed embryos is partially restored just prior to implantation.

### Downregulation of the 5-methylcytosine dioxygenase TET2 leads to DNA hypermethylation in embryos recovered from vitrification

**Fig. 2:**
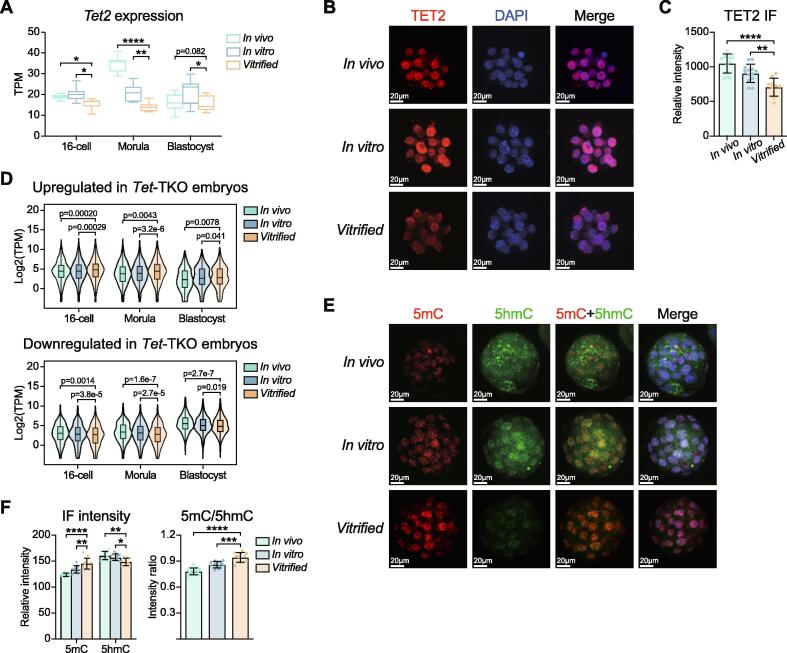
**Downregulation of the 5-methylcytosine dioxygenase TET2 leads to DNA hypermethylation in embryos recovered from vitrification.(A)** Box plots showing the expression level of Tet2 in all embryo mRNA-seq replicates (n = 8 for each sample). **(B)** IF images depicting the typical morphology of embryos stained for the TET2 protein at the morula stage. **(C)** Bar charts displaying the IF intensity of TET2 staining quantified by Imaris software. Each point represents the intensity of an individual embryo (n = 13 for *in vivo*, n = 13 for *in vitro* and n = 10 for vitrified). **(D)** Violin plots with embedded box plots showing the expression levels of Tet-sensitive genes at each stage. These genes were DEGs identified in E3.5 Tet-TKO mouse embryos compared to the WT control (n = 1738 upregulated genes and 1799 downregulated genes) according to the published data.[26] **(E)** IF images depicting the typical morphology of embryos stained for 5mC and 5hmC at the blastocyst stage. **(F)** Bar charts displaying the IF intensity of 5mC and 5hmC staining quantified by Imaris software, and the 5mC/5hmC ratios. Each point represents the intensity of an individual embryo (n = 13 for *in vivo*, n = 12 for *in vitro* and n = 10 for vitrified).

We then focused on the dysregulated genes which might be responsible for the developmental defects of the vitrified-thawed embryos. Differentially expressed gene (DEG) analysis revealed a considerable number of genes dysregulated at the morula and blastocyst stages in the *vitrified group* (Fig. S1E, F and Sheet 2–5 of Table S2). Dysregulated genes were implicated in biological processes including stress response, macromolecule metabolism, gene expression regulation, embryo development, cell cycle and apoptotic process (Fig. 1F and Fig. S1G). Interestingly, DNA methylation or demethylation was present in the enriched pathways among downregulated genes of the *vitrified group* at the blastocyst stage, and we observed that *Tet2*, the gene encoding a 5-methylcytosine (5mC) dioxygenase, was among the shared downregulated genes in morulae and blastocysts of the *vitrified group* (Fig. 1G). Actually, *Tet2* was consistently downregulated from 16-cell to the blastocyst stages in the vitrified-thawed embryos, especially at the morula stage (Fig. 2A). Immunofluorescence (IF) staining further verified the decreased level of TET2 protein in morulae of the *vitrified group* (Fig. 2B, C).

To check whether genes sensitive to insufficient *Tet* expression were also affected upon vitrification-thawing, we identified DEGs in *Tet* triple knockout (*Tet*-TKO) mouse embryos at embryonic day 3.5 (E3.5) based on the published data.[26] Indeed, a large portion of these genes were consistently dysregulated in the *vitrified group* at the morula stage, and the consistent expression differences diminished by the blastocyst stage (Fig. 2D and Fig. S2A, B). These results were expected, as *Tet2* expression in the *vitrified group* was most significantly downregulated at the morula stage (Fig. 2A).

We next identified the overlap between DEGs presented in E3.5 *Tet*-TKO embryos and blastocysts of the *vitrified group*. We found that 30 % of the downregulated genes and 22 % of the upregulated genes in the *vitrified group* overlapped with DEGs in *Tet*-TKO embryos, and the hypergeometric test revealed a statistically significant enrichment of the overlap (Fig. S2C). These results indicate that a considerable proportion of the abnormal gene expression in vitrified-thawed embryos is associated with *Tet2* insufficiency. We then focused on the shared DEGs in *Tet-*TKO embryos and vitrified-thawed embryos at the blastocyst stage. Functional enrichment analysis revealed that the downregulated genes were closely related to embryo development, whereas upregulated genes were associated with various processes including ribosome biogenesis, autophagy, RNA processing and catabolic process (Fig. S2D). These terms were similar to the enriched GO terms of the total DEGs in the *vitrified group* (Fig. 1F). It appears that many development-associated genes were consistently downregulated in both *Tet*-TKO embryos and vitrified-thawed embryos, indicating that similar developmental abnormalities occur upon *Tet* deficiency or vitrification.

TET dioxygenases iteratively oxidize 5mC into three different products, leading to active DNA demethylation, and 5-hydroxymethylcytosine (5hmC) is the first and most abundant oxidative product.[51,52] We thus checked the overall DNA methylation level by IF staining, and noticed a remarkable increase in the 5mC level and a decrease in the 5hmC level, with a corresponding upregulation of the 5mC/5hmC ratio in the *vitrified group* (Fig. 2E, F). Collectively, these results indicate that with the downregulation of *Tet2* upon vitrification-thawing, TET-sensitive genes are extensively dysregulated, and the global DNA methylation level is correspondingly increased.

### Restore of *Tet2* expression by OPA1 rescues TET2-regulated genes and the global DNA methylation level in vitrified-thawed embryos

**Fig. 3:**
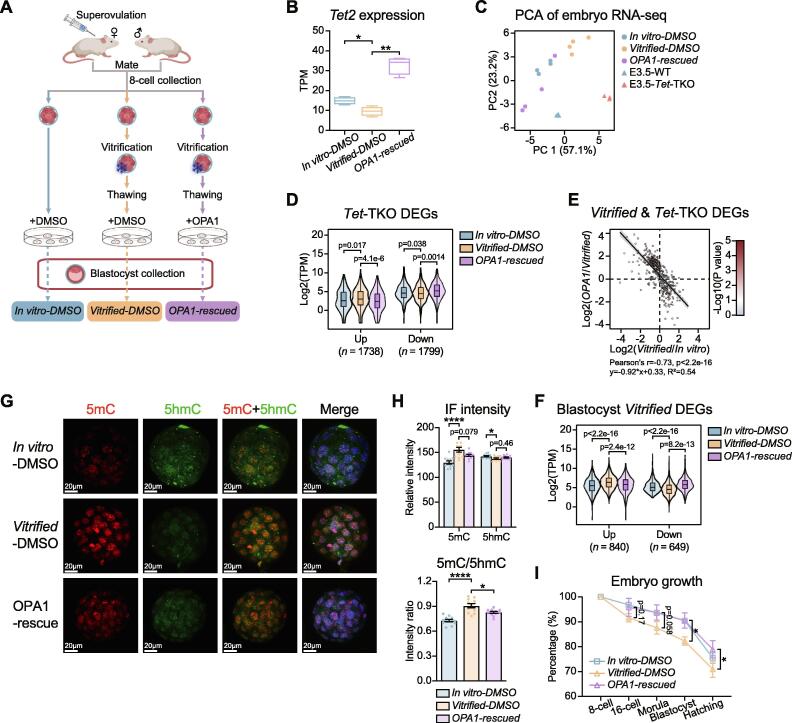
**Restore of Tet2 expression by OPA1 rescues TET2-regulated genes and the global DNA methylation level in vitrified-thawed embryos.(A)** Strategy for the OPA1 rescue experiments. The normal 8-cell embryos were collected and subjected to *in vitro* culture with 0.05 % DMSO in KSOM (*in vitro*-DMSO), or vitrification. After thawing of the vitrified embryos, the embryos were cultured with 0.05 % DMSO in KSOM (vitrified-DMSO) or 500 nM OPA1 (dissolved in 0.05 % DMSO) in KSOM (OPA1-rescued). At the blastocyst stage, embryos from the three groups were harvested for downstream analyses. **(B)** Box plots showing the expression level of Tet2 gene in all blastocyst mRNA-seq replicates of the drug-treated groups. **(C)** PCA of blastocyst mRNA-seq replicates of the drug-treated groups and the published data of E3.5 WT/Tet-TKO embryos[26] showing top two principal components that contribute to variance. PCA was performed with the expression levels of DEGs identified in E3.5 Tet-TKO embryos compared to E3.5 wt embryos (n = 3537 genes). **(D, F)** Violin plots with embedded box plots showing the expression levels of DEGs identified in E3.5 Tet-TKO embryos (D) and DEGs identified in blastocysts of the vitrified group (F) in the drug-treated groups at the blastocyst stage. Up, upregulated. Down, downregulated. **(E)** Scatter plots showing the fold change of the shared DEGs in E3.5 Tet-TKO embryos and blastocysts of the vitrified group (n = 393 genes). Each point represents the fold change of gene expression, with the x-axis representing log2(vitrified-DMSO/*in vitro*-DMSO) and the y-axis representing log2(OPA1-rescued/vitrified-DMSO). The color of each point represents the statistical significance (P value) of differential expression between the OPA1-rescued and vitrified-DMSO groups, as calculated using the limma-voom framework with an empirical Bayes moderated *t*-test in R. **(G)** IF images depicting the typical morphology of embryos stained for 5mC and 5hmC in the drug-treated groups at the blastocyst stage. **(H)** Bar charts displaying the IF intensity of 5mC and 5hmC staining quantified by Imaris software, and the 5mC/5hmC ratios. Each point represents the intensity of an individual embryo (n = 11 for each group). **(I)** Line charts displaying the embryo development rate at each stage for the drug-treated groups. Three biological replicates were tested for each group, with each replicate consisting of at least 30 initial embryos (see Sheet 1 of Table S1 for details).

We next sought to restore *Tet2* expression in vitrified-thawed embryos and treated these embryos immediately after thawing with OPA1 (Fig. 3A), a compound that has been demonstrated to enhance *Tet2* transcription.[53] We performed transcriptomic analysis and confirmed an elevated expression level of *Tet2* in OPA1-rescued embryos (Fig. 3B and Fig. S3A). PCA based on the expression levels of DEGs identified in E3.5 *Tet*-TKO embryos showed that vitrified-thawed embryos clustered closer to *Tet*-TKO embryos along PC1 (the principal component explaining the greatest variance), whereas OPA1-rescued embryos shifted in the opposite direction (Fig. 3C). We next focused on the shared DEGs in *Tet-*TKO embryos and vitrified-thawed embryos at the blastocyst stage, and compared their expression trends across each group. Notably, these genes exhibited similar aberrant expression trends in *Tet*-TKO embryos and vitrified-thawed embryos, and their expression levels were largely restored in OPA1-rescued embryos (Fig. 3D and Fig. S3B, C). We also evaluated the extent to which the expression of these DEGs was rescued, and found that the majority of them exhibited significantly corrected expression levels in OPA1-rescued embryos compared to vitrified-thawed embryos (Fig. 3E and Fig. S3D). Additionally, DEGs defined in blastocysts of the *vitrified group* (Fig. S1F) also exhibited restored expression levels in OPA1-rescued embryos (Fig. 3F and Fig. S3E, F). We validated the expression patterns of the shared DEGs associated with essential biological processes, and found that both the downregulated genes related to embryo development and the upregulated genes related to catabolic processes exhibited comparable expression levels in OPA1-rescued embryos and the *in vitro group* (Fig. S3G).

We then performed IF staining to check the overall DNA methylation level, and found that the amount of 5mC and 5hmC as well as the 5hmC/5mC ratio were all significantly restored in OPA1-rescued embryos at the blastocyst stage (Fig. 3G, H). Importantly, OPA1 treatment might also compensate for the restricted development potential of the vitrified-thawed embryos, as the rates of embryos reaching the morula and blastocyst stages were both increased (Fig. 3I and Sheet 1 of Table S1). Taken together, our results demonstrate that vitrified-thawed embryos recapitulated a subset of gene expression abnormalities induced by *Tet* depletion, and these abnormalities are closely associated with the early development. Meanwhile, OPA1 treatment restored expression levels for the majority of these DEGs in vitrified-thawed embryos, suggesting that the *Tet2* gene is a potential therapeutic target for improving the developmental competence of vitrified-thawed embryos.

### Promoters of lipid metabolism-related genes exhibit hypermethylation in blastocysts recovered from vitrified embryos

**Fig. 4:**
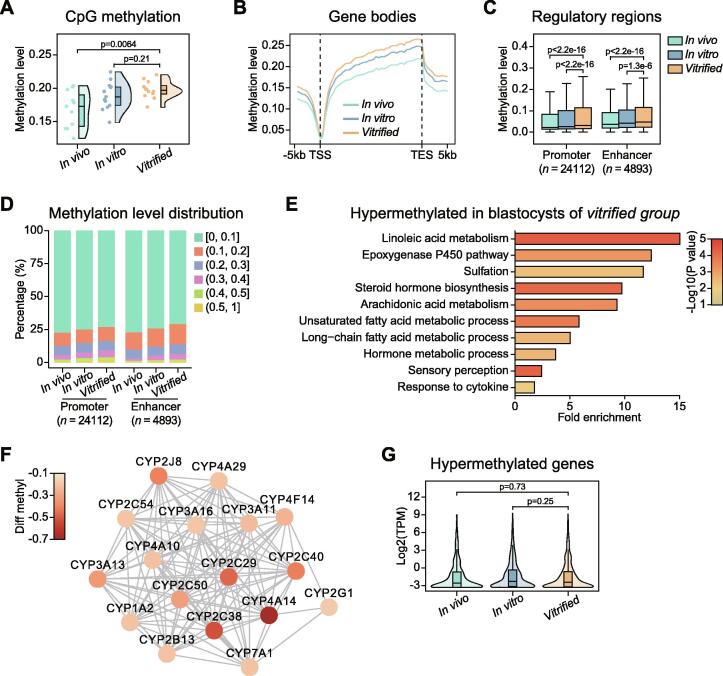
**Promoters of lipid metabolism-related genes exhibit hypermethylation in blastocysts recovered from vitrified embryos. (A)** Raincloud plots showing the genome-wide CpG methylation levels at the blastocyst stage. The genome-wide methylation level for each replicate was estimated by calculating the average CpG methylation per read and then the mean methylation across all reads. Each point corresponds to an individual replicate (*n* = 11 for *in vivo*, *n* = 13 for *in vitro* and *n* = 12 for *vitrified*). **(B)** Profiles depicting the CpG methylation levels across 5 kb upstream of the transcription start sites (TSSs) and 5 kb downstream of the transcription end sites (TESs) for all RefSeq genes at the blastocyst stage. Each gene body was divided into 40 intervals, while the upstream and downstream regions were each divided into 10 intervals (*n* = 28699 gene bodies). The methylation levels of these intervals were then calculated, and the replicate data were merged for each sample prior to plotting. **(C)** Box plots showing the methylation levels across promoter and enhancer regions at the blastocyst stage. The average CpG methylation level of each region was calculated for each group. **(D)** Stacked bar charts displaying the distribution of CpG methylation levels across enhancer regions and promoter regions of all RefSeq genes at the blastocyst stage. The methylation level of an individual region was calculated by dividing the number of methylated CpG sites by the total number of CpG sites within that region. **(E)** Horizontal bar charts displaying the top enriched functional categories for genes with hypermethylated promoters in the *vitrified group* compared to the *in vitro group* at the blastocyst stage. These promoters are labeled in Fig. S4J. **(F)** Network diagram provided by the Cytoscape software illustrating the interactions between proteins encoded by CYP genes with hypermethylated promoters in the *vitrified group* at the blastocyst stage. The color scale represents the differences in methylation levels (Diff methyl = methylation level [*vitrified group*] - methylation level [*in vitro group*]). **(G)** Violin plots with embedded box plots showing the expression levels of genes with hypermethylated promoters in the *vitrified group* at the blastocyst stage (*n* = 551 genes).

The significant downregulation of *Tet2* at the morula stage is expected to impede the ongoing demethylation process, resulting in pronounced differences in the DNA methylome at the blastocyst stage. Also, as the blastocyst is the last pre-implantation stage and possesses a hypomethylated genome,[17] methylation differences observed at this stage are of particular biological significance. Therefore, we collected embryos of each group at the blastocyst stage and performed low-input bisulfite sequencing[38] (LI-BS) to figure out the detailed alterations in the DNA methylome upon vitrification-thawing. The normal diploid copy numbers and high bisulfite conversion rates confirmed the high quality of the data, and uniform manifold approximation and projection (UMAP) analysis showed the presence of differences between groups (Fig. S4A–C). Coincident with the elevated 5mC level (Fig. 2E, F), the genome-wide CpG methylation level was upregulated in the *vitrified group* (Fig. 4A and Fig. S4D, E). Consistently, gene bodies as well as regulatory elements of genes were also hypermethylated in the *vitrified group* (Fig. 4B, C and Fig. S4F). Meanwhile, the methylation level distribution showed that the proportion of low methylated (methylation level < 0.3) promoters and enhancers was lower in the *vitrified group* (Fig. 4D), indicating that the DNA demethylation in gene regulatory regions was incomplete. These observations suggest that the global DNA demethylation process is disrupted in vitrified-thawed embryos. It has been reported that methylation levels at non-CpG sites remain relatively stable throughout mammalian pre-implantation development, which is in sharp contrast to the dramatic methylation changes observed at CpG sites.[17,21,54,55] In our case, the methylation levels at CHH (where H = A, T, or C) and CHG sites showed no significant differences among the groups (Fig. S4G), suggesting the differential response to vitrification between CpG and non-CpG sites.

We then calculated differentially methylated regions (DMRs; see Methods), and identified 4882 hypermethylated regions and 2528 hypomethylated regions in the *vitrified group* compared with the *in vitro group* (Fig. S4H and Sheet 1 of Table S3). Hypermethylated DMRs were consistently more abundant than hypomethylated DMRs across gene-associated elements, and the majority of DMRs were distributed in introns and intergenic regions (Fig. S4I). To identify more genes potentially affected by the incomplete DNA demethylation, we calculated the average methylation level within the promoter region of each gene (transcription start site ± 1 kb) and defined 551 hypermethylated promoters in the *vitrified group* (Fig. S4J and Sheet 2 of Table S3). Notably, genes with hypermethylated promoters were closely associated with metabolic processes, especially fatty acid-related processes, and several genes encoding cytochrome P450 enzymes (CYPs) associated with arachidonic acid metabolism[56] were present (Fig. 4E, F). Nevertheless, most of these hypermethylated genes were barely expressed at the blastocyst stage, and the hypermethylated CYP genes consistently maintained low expression levels from the 16-cell to the blastocyst stages (Fig. 4G and Fig. S4K), indicating that these lipid-related genes might not yet begin to play their biological roles during early development. Taken together, vitrification-thawing leads to extensive upregulation of DNA methylation levels, and functions of genes with hypermethylated promoters are enriched in lipid metabolism-related pathways. Although these genes may not be functional during pre-implantation stages, the aberrant DNA methylation modifications could persist into later developmental stages and/or affect other epigenetic states, thereby altering gene transcription activities and potentially leading to unexpected effects on related biological processes.

### The offspring derived from vitrified-thawed embryos display disturbances in glucose and lipid metabolism

**Fig. 5:**
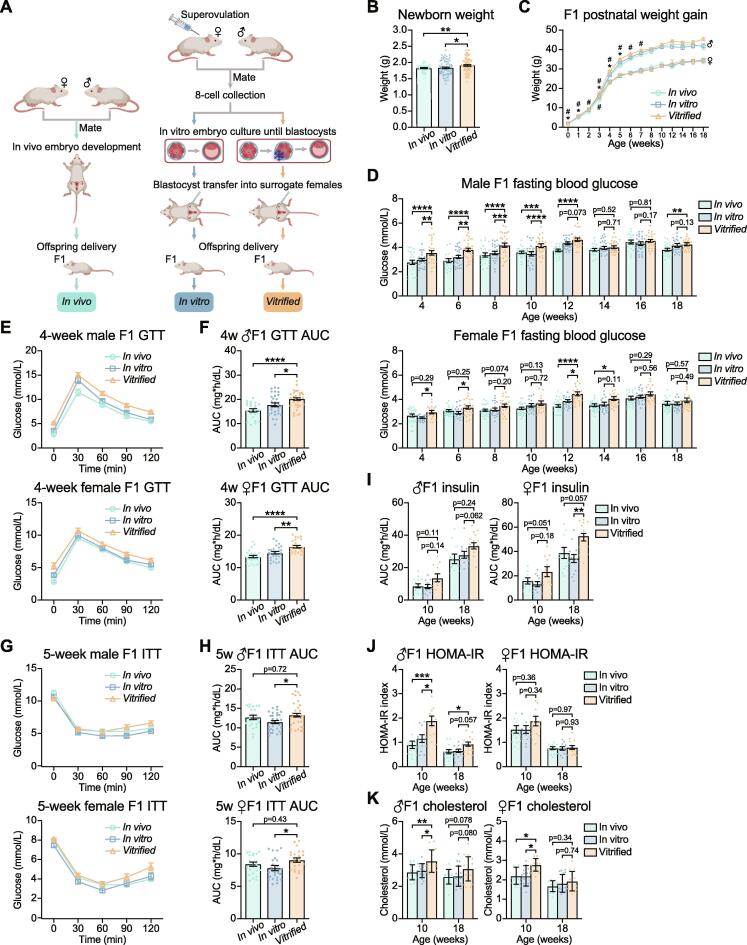
**The offspring derived from vitrified-thawed embryos display disturbances in glucose and lipid metabolism. (A)** Strategy for obtaining F1 offspring derived from embryos of each group. Blastocysts from each group were transferred into the uterine of pseudopregnant surrogate female mice, and the resulting offspring were referred to as the F1 generation. **(B)** Bar charts displaying the body weight of newborn offspring (*n* = 48 for *in vivo*, *n* = 71 for *in vitro* and *n* = 92 for *vitrified*). **(C)** Line charts displaying the body weight of offspring at the indicated weeks of age. The male and female offspring were measured separately after 3 weeks of age (*n* = 25 males and 25 females for *in vivo*, *n* = 38 males and 32 females for *in vitro*, *n* = 41 males and 36 females for *vitrified*). * indicates significant differences (P value < 0.05) between the *vitrified group* and the *in vitro group*, while # indicates significant differences between the *vitrified group* and the *in vivo group*. Non-significant differences were not labeled. **(D)** Bar charts displaying the blood glucose levels of male (upper panel) and female (lower panel) offspring after 16-h fasting at the indicated weeks of age (*n* = 23 males and 25 females for *in vivo*, *n* = 25 males and 20 females for *in vitro*, *n* = 26 males and 20 females for *vitrified*). **(E, G)** Line charts displaying the blood glucose levels of 4-week-old offspring during the GTT (E) and 5-week-old offspring during the ITT (G), with males (upper panels) and females (lower panels) measured separately. In each test, *n* = 23 males and 24 females for *in vivo*, *n* = 30 males and 26 females for *in vitro*, *n* = 30 males and 25 females for *vitrified*. **(F, H)** Bar charts displaying the AUC of the glucose levels measured during the GTT for 4-week-old offspring (4w F1; F) and ITT for 5-week-old offspring (5w F1; H). The male (upper panels) and female (lower panels) offspring were measured separately. **(I–K)** Bar charts displaying the serum insulin levels (I), HOMA-IR indices (J) and serum cholesterol levels (K; *n* = 14 males and 12 females for *in vivo*, *n* = 16 males and 15 females for *in vitro*, *n* = 14 males and 13 females for *vitrified*.) of offspring after 4-h fasting at the indicated weeks of age, with males (left panels) and females (right panels) measured separately. For insulin levels and HOMA-IR indices, *n* = 12 for each sex in each group. For the plots in (B, D, F and H–K), each point represents the value of an individual offspring.

Considering that genes with hypermethylated promoters in the *vitrified group* were involved in metabolism-related regulation and may function in the long-term health, we next focused on the postnatal growth of offspring, namely the F1 generation of each group (Fig. 5A and Sheet 2 of Table S1). Notably, birth weight in the *vitrified group* was increased compared to the control groups, and the elevation in body weight of male offspring persisted until week 18 (Fig. 5B, C). Moreover, body composition in the *vitrified group* was also slighted altered, with an increased body fat percentage and a decreased lean mass ratio (Fig. S5A).

We speculated that the weight gain observed in the *vitrified group* might relate to disturbed glucose metabolism, and measured representative glucose metabolic parameters in offspring from each group. Interestingly, the fasting blood glucose levels of offspring were higher in the *vitrified group* compared to the control, particularly in male offspring before week 12 (Fig. 5D). Meanwhile, the levels of glycated serum protein (GSP), reflecting the average blood glucose concentration in the past 1–3 weeks, was slightly elevated in the *vitrified group* compared to the *in vitro group* (Fig. S5B). Importantly, the glucose tolerance test (GTT) revealed that the blood glucose was elevated to a higher level after glucose injection and the glucose disposal was less effectively in the *vitrified group* compared to the control at week 4 (Fig. 5E, F), suggesting an impaired glucose tolerance. This glucose intolerance persisted until week 8 and tended to alleviate with subsequent development (Fig. S5C, D). To deduce whether the inefficient blood glucose consumption was caused by insulin resistance, we performed insulin tolerance test (ITT) and found that the response to insulin injection was weaker in the *vitrified group* compared to the *in vitro group* at week 5 through calculating the area under the curve (AUC) of blood glucose levels (Fig. 5G, H). Similarly, a weakened response to insulin was observed in offspring of the *vitrified group*, though it was less pronounced at an older age (Fig. S5E, F). Consistent with the insulin resistance phenotype, fasting serum insulin levels in the *vitrified group* were also elevated, and the homeostatic model assessment for insulin resistance (HOMA-IR) revealed significantly increased indices in the *vitrified group* compared to the control (Fig. 5I, J). In addition, offspring of the *vitrified group* showed a higher level of fasting serum cholesterol, particularly at a younger age (Fig. 5K), whereas the levels of triglyceride and low-density lipoprotein-cholesterol (LDL-C) in serum were comparable between each group (Fig. S5G, H). Collectively, vitrification-thawing of mouse pre-implantation embryos increases the risk of glucose and lipid metabolism disturbances in the early postnatal life, and the abnormalities tend to alleviate as the offspring grew older.

### Hepatic tissues of the adult offspring derived from vitrified-thawed embryos exhibit lipid deposition and mitochondrial dysfunction

**Fig. 6:**
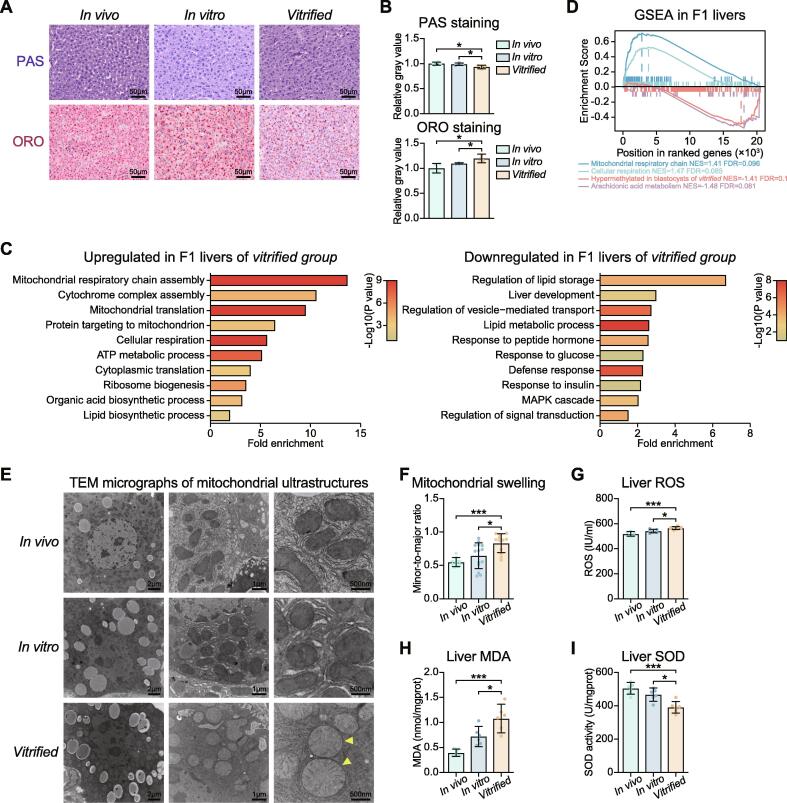
**Hepatic tissues of the adult offspring derived from vitrified-thawed embryos exhibit lipid deposition and mitochondrial dysfunction. (A)** Bright-field images depicting the typical morphology of hepatic tissues from 10-week-old female offspring after PAS staining (upper panels) and ORO staining (lower panels). **(B)** Bar charts displaying the mean gray values which quantify the glycogen storage (upper panel) and integrated density gray values which quantify the lipid droplets (lower panel) measured by ImageJ software. Each point represents the value of an individual replicate (*n* = 4 for each group). **(C)** Horizontal bar charts displaying the top enriched functional categories for upregulated genes (upper panel) and downregulated genes (lower panel) identified in the livers of 10-week-old female offspring from the *vitrified group* compared to the *in vitro group*. **(D)** GSEA enrichment plots showing the dysregulation of the indicated gene sets in the *vitrified group* in livers of 10-week-old female offspring. The y-axis represents the running enrichment scores (ESs; *vitrified group* compared to *in vitro group*) provided by GSEA software. **(E)** TEM images depicting the typical morphology of mitochondria in the livers of 10-week-old female offspring. Yellow arrows highlight the typical mitochondrial swelling. **(F)** Bar charts displaying the minor-to-major axis ratios of mitochondria in the livers of 10-week-old female offspring measured using ImageJ software. Each point represents the value of an individual mitochondrion (*n* = 10 for *in vivo*, *n* = 14 for *in vitro* and *n* = 12 for *vitrified*). **(G–I)** Bar charts displaying the levels of ROS (G), MDA (H) and SOD (I) in the liver tissues of 10-week-old female offspring. Each point represents the value of an individual replicate (*n* = 6 for each group in each measurement).

As the primary site in the body for carbohydrate and lipid biosynthesis, the liver is essential for maintaining normal glucose and lipid homeostasis.[57,58] We thus focused on hepatic functions and collected liver tissues of 10-week-old female offspring for histological analyses. We found that glycogen storage was reduced and lipid droplets were more severely accumulated in the livers of the *vitrified group* compared to the control, as demonstrated by Periodic Acid-Schiff (PAS) staining and Oil Red O (ORO) staining, respectively (Fig. 6A, B). These findings suggest hepatic disturbances in carbohydrate metabolism and lipid synthesis in the offspring from vitrified-thawed embryos.

To investigate the underlying mechanism of these metabolic disturbances, we next performed transcriptomic profiling for livers of 10-week-old female offspring from each group (Fig. S6A, B). Notably, a considerable number of metabolism-associated genes were dysregulated in the *vitrified group* revealed by DEG analysis. Many downregulated genes were involved in lipid metabolic process, response to insulin and liver development, and upregulated genes were closely associated with lipid biosynthetic process and mitochondria-related respiratory functions (Fig. S6C and Fig. 6C). The extensive dysregulation of genes associated with fatty acid metabolism as well as mitochondrial respiration was further validated through the gene set enrichment analysis (GSEA; Fig. 6D). To detect mitochondrial defects suggested by the transcriptomic data in hepatic tissues of the *vitrified group*, we captured the ultrastructure of livers from 10-week-old offspring for each group through transmission electron microscopy. Interestingly, we observed abnormal mitochondrial swelling in the *vitrified group* (Fig. 6E, F), supporting the dysfunction of mitochondrial respiration. Moreover, the level of reactive oxygen species (ROS) in hepatic tissues of the *vitrified group* was also elevated (Fig. 6G), indicating the imbalance of ROS production and elimination, which might lead to oxidative stress and further exacerbate mitochondrial dysfunctions.[59] Meanwhile, the final product of arachidonic acid peroxidation, malondialdehyde (MDA),[60] was also increased in the *vitrified group p* (Fig. 6H), indicating a higher degree of ROS attack and elevated oxidative stress. In addition, we detected reduced activity of superoxide dismutase (SOD) in the *vitrified group*, and comparable activities of other enzymes responsible for ROS removal including glutathione peroxidase (GSH-PX) and Catalase (CAT; Fig. 6I and Fig. S6D, E). SOD catalyzes the conversion of superoxide to oxygen and hydrogen peroxide[61], and these results suggest that the impaired SOD-mediated ROS elimination may contribute to the elevated oxidative stress in livers of the *vitrified group*. Taken together, hepatic fatty acid and oxidation–reduction homeostasis is disturbed in mouse offspring derived from vitrified-thawed embryos, resulting in lipid deposition and mitochondrial dysfunctions in hepatic tissues, and these defects may be attributed to dysregulation of genes involved in lipid metabolism and cellular respiration.

### Metabolism-associated genes are differentially expressed in offspring derived from vitrified-thawed embryos despite their normal DNA methylation levels

**Fig. 7:**
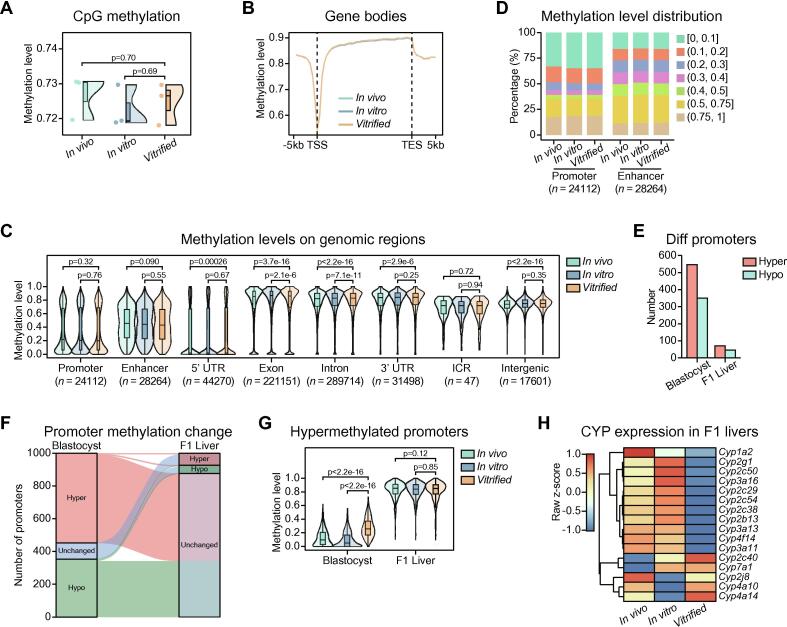
**Metabolism-associated genes are differentially expressed in offspring derived from vitrified-thawed embryos despite their normal DNA methylation levels (A)** Raincloud plots showing the genome-wide CpG methylation levels in the livers of 10-week-old female offspring. Each point corresponds to an individual replicate (*n* = 3 for each group). **(B)** Profiles depicting the CpG methylation levels across 5 kb upstream of TSSs and 5 kb downstream of the TESs for all RefSeq genes (*n* = 28699 gene bodies) in the livers of 10-week-old female offspring. **(C)** Violin plots with embedded box plots showing the methylation levels across the indicated genomic elements in the livers of 10-week-old female offspring. **(D)** Stacked bar charts displaying the distribution of CpG methylation levels across enhancer regions and promoter regions of all RefSeq genes in the livers of 10-week-old female offspring. **(E)** Bar charts displaying the numbers of differentially methylated promoters identified in the *vitrified group* compared to the *in vitro group* in blastocysts and offspring’s livers. These promoters are labeled in Fig. S4J and S7G. Diff, differentially methylated. Hyper, hypermethylated. Hypo, hypomethylated. **(F)** Alluvial diagram illustrating the track of differentially methylated promoters identified in the *vitrified group* from blastocyst samples to liver samples. Only the promoters exhibiting differential methylation in blastocysts (left column) or offspring’s livers (right column) of the *vitrified group*, as compared to the *in vitro group*, were included in the plots. **(G)** Violin plots with embedded box plots showing the methylation levels of specific promoter regions in blastocysts and offspring’s livers. These promoters were hypermethylated in the *vitrified group* at the blastocyst stage (*n* = 551 promoters), as labeled in red in Fig. S4J. **(H)** Heatmap depicting the relative expression levels of specific CYP genes in the livers of 10-week-old female offspring. The promoter regions of these genes were hypermethylated in the *vitrified group* compared to the *in vitro group* at the blastocyst stage. Hierarchical clustering was performed on rows. For the plotting in (A–D), see Fig. 4A–D for detailed explanations.

We then investigated whether the aberrant DNA methylation detected in blastocysts upon vitrification-thawing was inherited in the postnatal life, and profiled DNA methylation landscape in livers of 10-week-old offspring for each group through whole-genome bisulfite sequencing (WGBS; Fig. S7A–C). Notably, as the genome becomes highly methylated in the terminally differentiated tissue, liver, global CpG methylation of the *vitrified group* recovered to a level comparable with the control groups, and the methylation levels on CHH and CHG sites were also comparable (Fig. 7A and Fig. S7D–F). Consistently, no significant alterations in DNA methylation were observed at gene bodies or regulatory elements in the *vitrified group* (Fig. 7B, C). Meanwhile, the proportion of low methylated (methylation level < 0.3) promoters and enhancers was equivalent in the *vitrified group* and the *in vitro group* (Fig. 7D). Consistently, only a small subset of genes possessed differentially methylated promoters in the *vitrified group*, and most of the hypermethylated promoters identified in blastocysts of the *vitrified group* showed no difference in the livers of the offspring (Fig. 7E–G and Fig. S7G, H). Notably, these hypermethylated promoters exhibited low methylation levels (< 0.3) at the blastocyst stage in the control groups, and were restored to similarly high methylation levels (> 0.8) in adult mice of all groups (Fig. 7G). These results suggest that as DNA methylation modifications were vitrified-thawed embryos globally re-established after implantation and reached a high level in the postnatal life, the aberrantly methylated sites detected in blastocysts recovered from vitrified embryos were also restored to a highly methylated status. Particularly, lipid metabolism-associated processes were among the top enriched pathways for hypermethylated genes in blastocysts of the *vitrified group* (Fig. 4E). While these hypermethylated genes were inherently lowly expressed at the blastocyst stage (Fig. 4G), they may become functional in postnatal metabolic processes. Intriguingly, these genes including multiple CYP genes were extensively downregulated in adult livers of the *vitrified group*, despite the normal DNA methylation levels on their promoters compared to the control groups (Fig. 7H and Fig. S7I, J). Collectively, our data suggest that most of the DNA methylation alterations present in vitrified-thawed embryos at pre-implantation stages have been corrected in the livers of adult offspring. However, these genes, which previously exhibit aberrant epigenetic marks, fail to maintain normal expression levels when their biological functions are required, thereby threatening long-term health after birth. Typically, the fatty acid metabolism-associated genes, which are barely expressed at the pre-implantation stages but play indispensable roles in metabolic processes in adult hepatic tissues, are downregulated in offspring from vitrified-thawed embryos, potentially accounting for the observed metabolic defects.

## Discussion

During the ART cycles, cryopreservation of human embryos at day 3 cleavage stage is widely performed and has been proved to result in higher live birth rates.[[62], [63], [64]] In our study, we selected 8-cell mouse embryos for vitrification as they most closely morphologically resemble day-3 human cleavage-stage embryos. This selection was based on the shared morphological characteristics between mouse and human 8-cell embryos, including enhanced cell packing, expanded membrane surface area, and reorganized cytoskeletal architecture. These morphological features directly affect cryoprotectant permeability, dehydration kinetics and susceptibility to chilling injury, thus critically impacting vitrification outcomes. Thus, we used 8-cell mouse embryos to mimic the structural conditions under which human embryos are vitrified. Nevertheless, it is noteworthy that the early developmental processes in mice and human are not exactly synchronous, and one of the main differences is the timing of zygotic genome activation (ZGA). ZGA is a critical process which marks the acquisition of totipotency and establishes the gene expression pattern required for continued development.[65,66] Human ZGA mainly occurs at the 8-cell stage, whereas the major wave of mouse ZGA occurs at the late 2-cell stage.[[67], [68], [69], [70]] Therefore, further investigations using embryos precisely at the wave of ZGA for vitrification are required to figure out how transcription activation is restored post-thawing.

While vitrification is fundamentally a physical process, cryoprotectant agents or the procedures may induce oxidative stress by increasing ROS levels.[35,[71], [72], [73]] Notably, multiple studies have revealed the downregulation of *Tet2* expression under oxidative stress,[[74], [75], [76], [77], [78]] suggesting *Tet2*′s particular susceptibility to vitrification-induced oxidative stress. Moreover, as an Fe(II)-dependent dioxygenase,[[79], [80], [81]] TET2 protein is potentially highly susceptible to ROS, which can oxidize the catalytic iron center of TET2 and inhibit its catalytic activity.[82,83] As TET2 can activate its own expression by interacting with the estrogen receptor ERα,[84] the inhibition of TET2 catalytic activity by ROS is likely to cause downregulation of the *Tet2* gene. Despite the above hypotheses regarding the rationale for the specific downregulation of *Tet2* upon vitrification-thawing, the precise underlying mechanism requires elucidation in future studies.

Our data provide consistent evidence for overall DNA hypermethylation, including the increased level of 5mC and the decreased level of 5hmC in vitrified-thawed embryos. While 5hmC is the first and most abundant oxidative product in the TET-catalyzed DNA demethylation pathway, 5hmC can be further oxidized iteratively to 5-formylcytosine (5fC) and 5-carboxylcytosine (5caC) by TET.[51,85] Although the 5fC and 5caC exhibit a very low abundance[51,52], emerging evidence indicates that they may also serve as functional epigenetic marks. A recent landmark study demonstrated that 5fC acts as an activating epigenetic mark that facilitates chromatin openness via promoting Pol III recruitment and tRNA transcription during ZGA in Xenopus and mouse embryos.[86,87] Considering the promotive role of 5fC towards ZGA, further studies are warranted to investigate alterations in 5fC levels and transcriptional activities of 5fC-marked loci in 2-cell mouse embryos following vitrification-thawing.

In our case, vitrification seems to induce a transient DNA hypermethylation in early embryos, as DNA methylation had been recovered to a normal level in livers of offspring derived from vitrified-thawed embryos. However, the metabolism-associated genes remain dysregulated despite the normal DNA methylation level, indicating that compensatory epigenetic mechanisms may contribute to the pathogenesis of metabolic disturbances induced by embryo vitrification. Actually, the time window for the dramatic epigenetic reprogramming during early embryo development is limited, and the delayed developmental progression in vitrified-thawed embryo may impede the remodeling processes, leading to irreversible epigenetic dysregulations. Such dysregulations may manifest in two aspects: firstly, the aberrant DNA methylation marks may disturb the post-implantation reestablishment of the methylome, which is essential for lineage specification during gastrulation; secondly, other epigenetic marks such as histone modifications may also be affected by DNA methylation and deliver inaccurate epigenetic information during prenatal and postnatal developmental stages. Therefore, future explorations are required to elucidate how the downstream effects of DNA hypermethylation perpetuate an aberrant chromatin state for metabolism-associated genes, thereby exerting potential adverse effects on long-term metabolic health.

## Conclusion

In conclusion, our study reveals that embryo vitrification disturbs pre-implantation DNA demethylation by reducing *Tet2* expression in mice, leading to postnatal metabolic disturbances with dysregulation of relevant genes in hepatic tissues. These findings highlight the epigenetic origin of metabolic risks associated with vitrification, and future efforts are required to explore therapeutic strategies based on targeting *Tet2* activity during vitrification to mitigate long-term metabolic risks. Further investigations on the alterations of other epigenetic modifiers in response to embryo vitrification are also warranted to explore more preventive interventions.

## Ethics statement

All animal experiments were approved by the ACUC of Zhejiang University School of Medicine (Approval NO. ZJU20200035) and performed in accordance with the guidelines of Institutional Animal Care and Use Committee (IACUC).

## Funding

This work was supported by National Key Research and Development Program of China (2022YFC2703500 to D.Z.), the National Natural Science Foundation of China (32200455 to C.C.), Zhejiang University Global Partnership Fund to D.Z., the Medical and Health Research Project of Zhejiang Province (2022RC200 to J.L.) and the Natural Science Foundation of Zhejiang Province (LTGY23H040007 to J.L.).

## Compliance with Ethics Requirements

All Institutional and National Guidelines for the care and use of animals (fisheries) were followed.

## Declaration of competing interest

The authors declare that they have no known competing financial interests or personal relationships that could have appeared to influence the work reported in this paper.
