# Supplementary material for: Embryo vitrification disturbs the pre-implantation DNA demethylation process and affects hepatic functions to threaten long-term metabolic health in mice
Source: J Adv Res. 2025 Nov 17;86:621–37. doi: 10.1016/j.jare.2025.11.032 (PMC13453966; doi:10.1016/j.jare.2025.11.032)
Supplement: Supplementary file 1 [file mmc4.pdf]

Figure S1

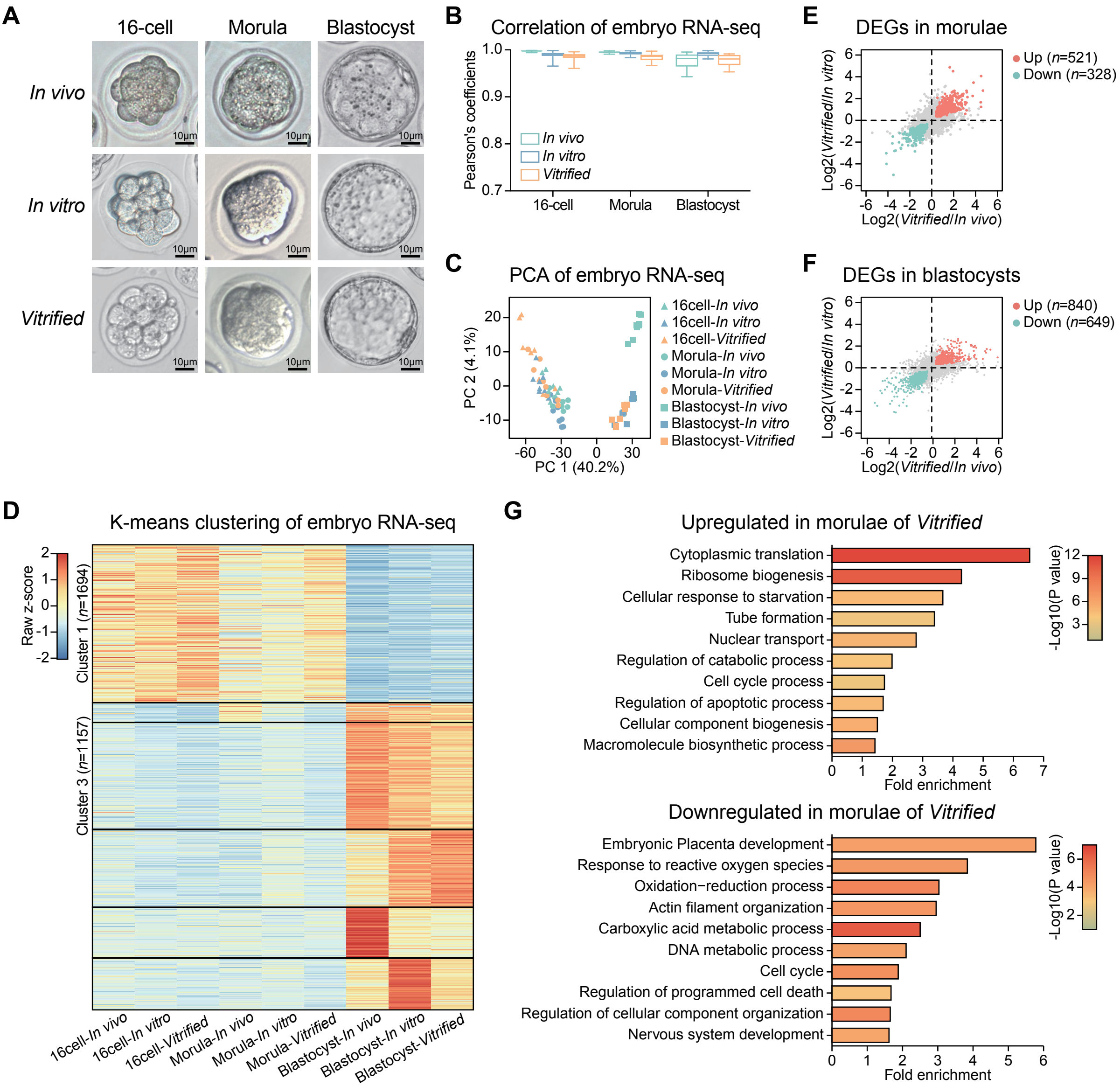
A
16-cell
Morula
Blastocyst
In vivo
In vitro
Vitrified
10μm
B
Correlation of embryo RNA-seq
Pearson's coefficients
In vivo
In vitro
Vitrified
16-cell
Morula
Blastocyst
C
PCA of embryo RNA-seq
PC 2 (4.1%)
PC 1 (40.2%)
16cell-In vivo
16cell-In vitro
16cell-Vitrified
Morula-In vivo
Morula-In vitro
Morula-Vitrified
Blastocyst-In vivo
Blastocyst-In vitro
Blastocyst-Vitrified
E
DEGs in morulae
Up (n=521)
Down (n=328)
Log2(Vitrified/In vitro)
Log2(Vitrified/In vivo)
F
DEGs in blastocysts
Up (n=840)
Down (n=649)
Log2(Vitrified/In vitro)
Log2(Vitrified/In vivo)
D
K-means clustering of embryo RNA-seq
Raw z-score
Cluster 1 (n=1694)
Cluster 3 (n=1157)
16cell-In vivo
16cell-In vitro
16cell-Vitrified
Morula-In vivo
Morula-In vitro
Morula-Vitrified
Blastocyst-In vivo
Blastocyst-In vitro
Blastocyst-Vitrified
G
Upregulated in morulae of Vitrified
Cytoplasmic translation
Ribosome biogenesis
Cellular response to starvation
Tube formation
Nuclear transport
Regulation of catabolic process
Cell cycle process
Regulation of apoptotic process
Cellular component biogenesis
Macromolecule biosynthetic process
Fold enrichment
-Log10(P value)
Downregulated in morulae of Vitrified
Embryonic Placenta development
Response to reactive oxygen species
Oxidation−reduction process
Actin filament organization
Carboxylic acid metabolic process
DNA metabolic process
Cell cycle
Regulation of programmed cell death
Regulation of cellular component organization
Nervous system development
Fold enrichment
-Log10(P value)

Figure S1. Analyses of mouse embryo mRNA-seq data (corresponds to Figure 1).
(A) Bright-field images depicting the typical morphology of harvested embryos for each stage.
(B) Box plots showing the pairwise Pearson's correlation coefficients calculated between all replicates for each sample (n = 8) in the embryo mRNA-seq dataset. The data matrix containing TPM of all genes was used for this calculation.
(C) PCA of all embryo mRNA-seq replicates showing top two principal components that contribute to variance. PCA was performed with the expression levels of top 3,000 genes showing significant changes along the embryo developmental trajectory.
(D) Heatmap depicting the k-means clustering (k = 6) of top 3000 significant genes identified by Monocle, based on their expression patterns in all embryo mRNA-seq samples. These genes are listed in Table S2.
(E, F) Scatter plots showing the expression fold changes for all genes at the morula stage (E) and the blastocyst stage (F). The x-axis represents the *vitrified group*/*in vivo group* ratios, and the y-axis represents the *vitrified group*/*in vitro group* ratios. The labeled points represent genes differentially expressed in *vitrified group* compared to both *in vitro group* and *in vivo group*, which were identified using edgeR and considered as confident DEGs of *vitrified group*, and the number of identified genes are indicated in the plots. Up, upregulated in *vitrified group*. Down, downregulated in *vitrified group*.
(G) Horizontal bar charts displaying the top enriched functional categories for upregulated genes (upper panel) and downregulated genes (lower panel) identified in *vitrified group* at the morula stage.

Figure S2

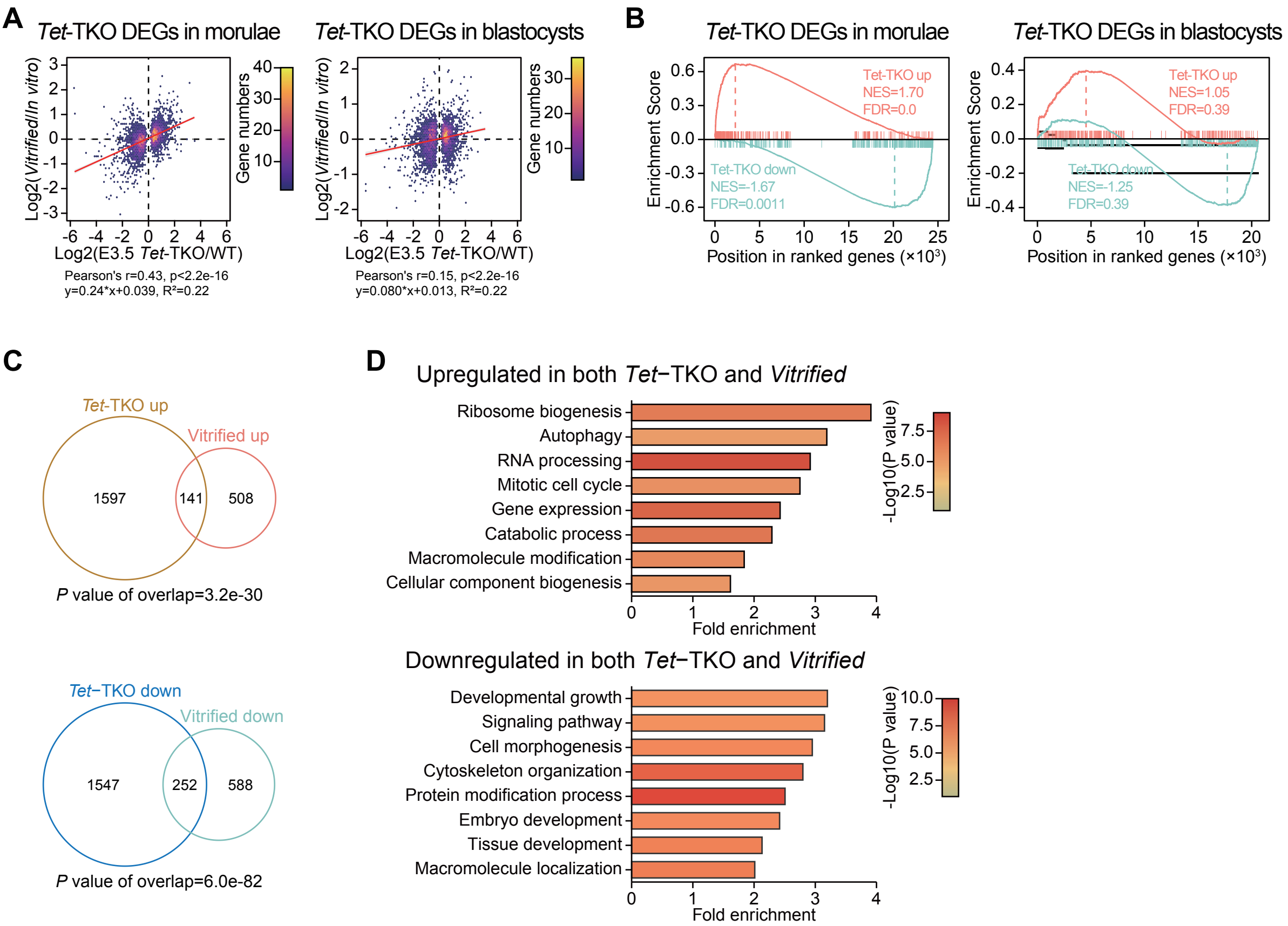

Figure S2. Comparison of gene expression in *Tet*-depleted embryos and vitrified-thawed embryos (corresponds to Figure 2).

(A) Scatter plots showing the fold change of DEGs identified in E3.5 *Tet*-TKO embryos (n = 3537 genes). Each point represents the fold change of an individual gene, with the x-axis representing log2(E3.5 *Tet*-TKO/E3.5 WT), and the y-axis representing log2(*vitrified group*/*in vitro group*) at the morula stage (left panel) and the blastocyst stage (right panel). The color of each point reflects the density of gene numbers.

(B) GSEA enrichment plots showing the dysregulation of *Tet*-sensitive genes in *vitrified group* at the morula stage (left panel) and the blastocyst stage (right panel). These genes were DEGs identified in E3.5 *Tet*-TKO embryos.The y-axis represents the running enrichment scores (ESs; *vitrified group* compared to *in vitro group*).

(C) Venn diagrams illustrating the overlap between DEGs presented in E3.5 *Tet*-TKO embryos and blastocysts of the *vitrified group*. Statistical significance (P value) of the overlap was assessed using the hypergeometric test (implemented by the phyper function in R). Upregulated (upper panel) and downregulated genes (lower panel) were analyzed separately.

(D) Horizontal bar charts displaying the top enriched functional categories for shared upregulated genes (upper panel) and downregulated genes (lower panel) identified in both E3.5 *Tet*-TKO embryos and blastocysts of the *vitrified group*.

Figure S3

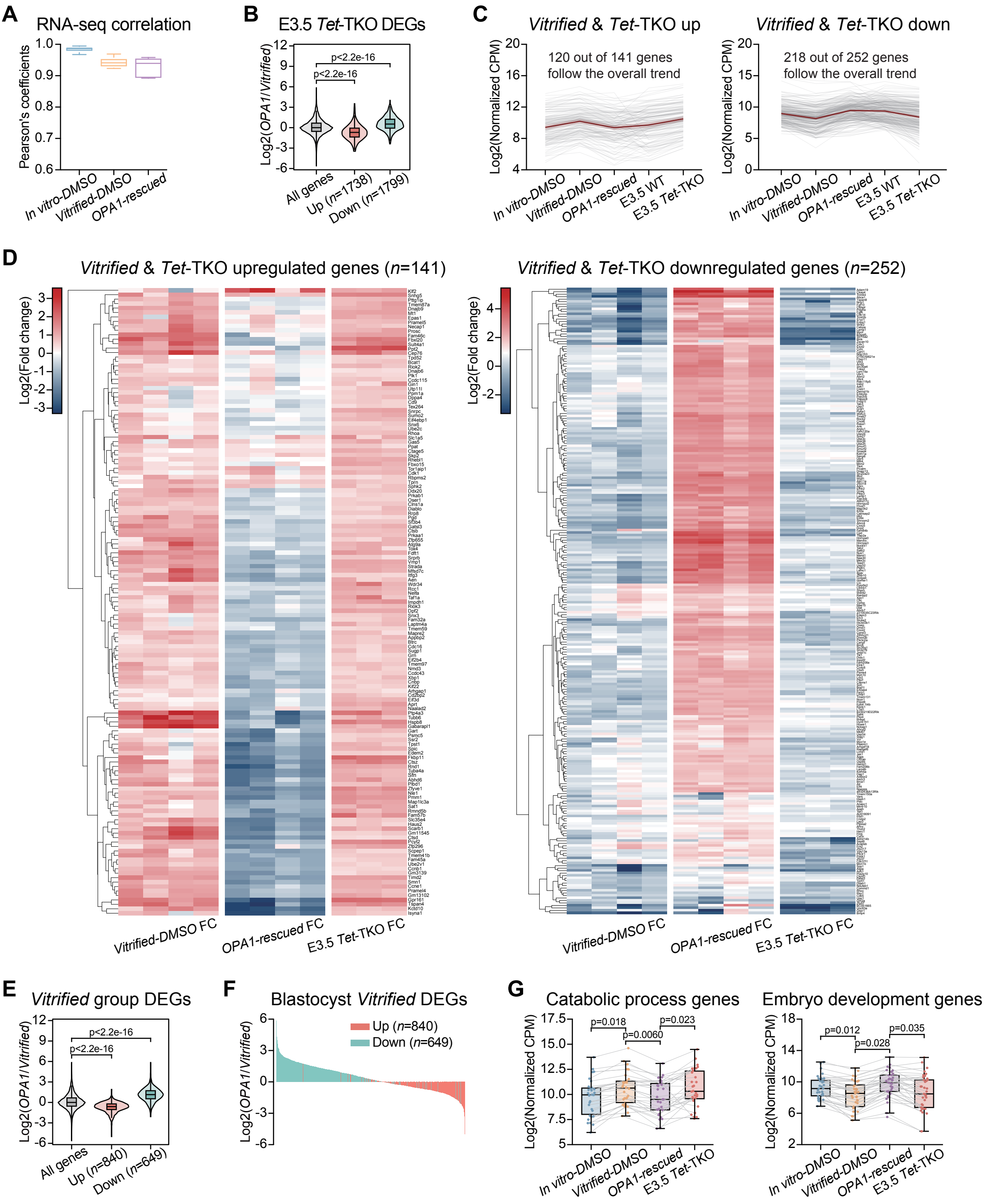
A
RNA-seq correlation
Pearson's coefficients
In vitro-DMSO
Vitrified-DMSO
OPA1-rescued
B
E3.5 Tet-TKO DEGs
Log2(OPA1/Vitrified)
p<2.2e-16
p<2.2e-16
All genes
Up (n=1738)
Down (n=1799)
C
Vitrified & Tet-TKO up
Log2(Normalized CPM)
120 out of 141 genes
follow the overall trend
In vitro-DMSO
Vitrified-DMSO
OPA1-rescued
E3.5 WT
E3.5 Tet-TKO
Vitrified & Tet-TKO down
Log2(Normalized CPM)
218 out of 252 genes
follow the overall trend
In vitro-DMSO
Vitrified-DMSO
OPA1-rescued
E3.5 WT
E3.5 Tet-TKO
D
Vitrified & Tet-TKO upregulated genes (n=141)
Log2(Fold change)
Vitrified-DMSO FC
OPA1-rescued FC
E3.5 Tet-TKO FC
Vitrified & Tet-TKO downregulated genes (n=252)
Log2(Fold change)
Vitrified-DMSO FC
OPA1-rescued FC
E3.5 Tet-TKO FC
E
Vitrified group DEGs
Log2(OPA1/Vitrified)
p<2.2e-16
p<2.2e-16
All genes
Up (n=840)
Down (n=649)
F
Blastocyst Vitrified DEGs
Log2(OPA1/Vitrified)
Up (n=840)
Down (n=649)
G
Catabolic process genes
Log2(Normalized CPM)
p=0.018
p=0.0060
p=0.023
In vitro-DMSO
Vitrified-DMSO
OPA1-rescued
E3.5 Tet-TKO
Embryo development genes
Log2(Normalized CPM)
p=0.012
p=0.028
p=0.035
In vitro-DMSO
Vitrified-DMSO
OPA1-rescued
E3.5 Tet-TKO

Figure S3. Analyses of OPA1-rescued embryos (corresponds to Figure 3).
(A) Box plots showing the pairwise Pearson's correlation coefficients calculated between all replicates for each sample (*n* = 4) in the blastocyst mRNA-seq dataset of the drug-treated groups. The data matrix containing TPM of all genes was used for this calculation.
(B, E) Violin plots with embedded box plots showing the fold change of DEGs identified in E3.5 *Tet*-TKO embryos (B) and DEGs identified in blastocysts of the *vitrified group* (E) in the drug-treated groups at the blastocyst stage. Up, upregulated. Down, downregulated.
(C) Trajectory profiles illustrating the expression trends of genes in the drug-treated groups at the blastocyst stage and the published data of E3.5 WT/*Tet*-TKO embryos. These genes are shared upregulated genes (up; left panel) and downregulated genes (down; right panel) in E3.5 *Tet*-TKO embryos and blastocysts of the *vitrified group.* Gray lines represent individual gene trajectories, and the robust trend line in dark red represents the mean expression level of all analyzed genes in each group, with the 95% confidence interval shaded around the mean line.
(D) Heatmap depicting the fold change of shared upregulated genes (up; left panel) and downregulated genes (down; right panel) in E3.5 *Tet*-TKO embryos and blastocysts of the *vitrified group. Vitrified-DMSO* FC represents the *vitrified-DMSO group*/*in vitro-DMSO group* ratios, *OPA1-rescued* FC represents the *OPA1-rescued group*/*vitrified-DMSO group* ratios, and E3.5 *Tet*-TKO FC represents the E3.5 *Tet*-TKO/E3.5 WT ratios. Red indicates the positive fold change, whereas blue represents the negative fold change. Each column represents an individual replicate, and hierarchical clustering was performed on rows. FC, fold change.
(F) Bar charts displaying the fold change of DEGs identified in blastocysts of the *vitrified group*. Bars are sorted by the *OPA1-rescued group*/*vitrified-DMSO group* ratios in descending order.
(G) Box plots showing the expression levels of genes associated with catabolic processes (left panel; *n* = 30) and embryo development (right panel; *n* = 37) in the drug-treated groups at the blastocyst stage and the published data of E3.5 *Tet*-TKO embryos. These genes have been identified as shared DEGs in E3.5 *Tet*-TKO embryos and blastocysts of the *vitrified group.* Each point represents the expression level of an individual gene, and gray lines represent the individual gene trajectories across groups.

Figure S4

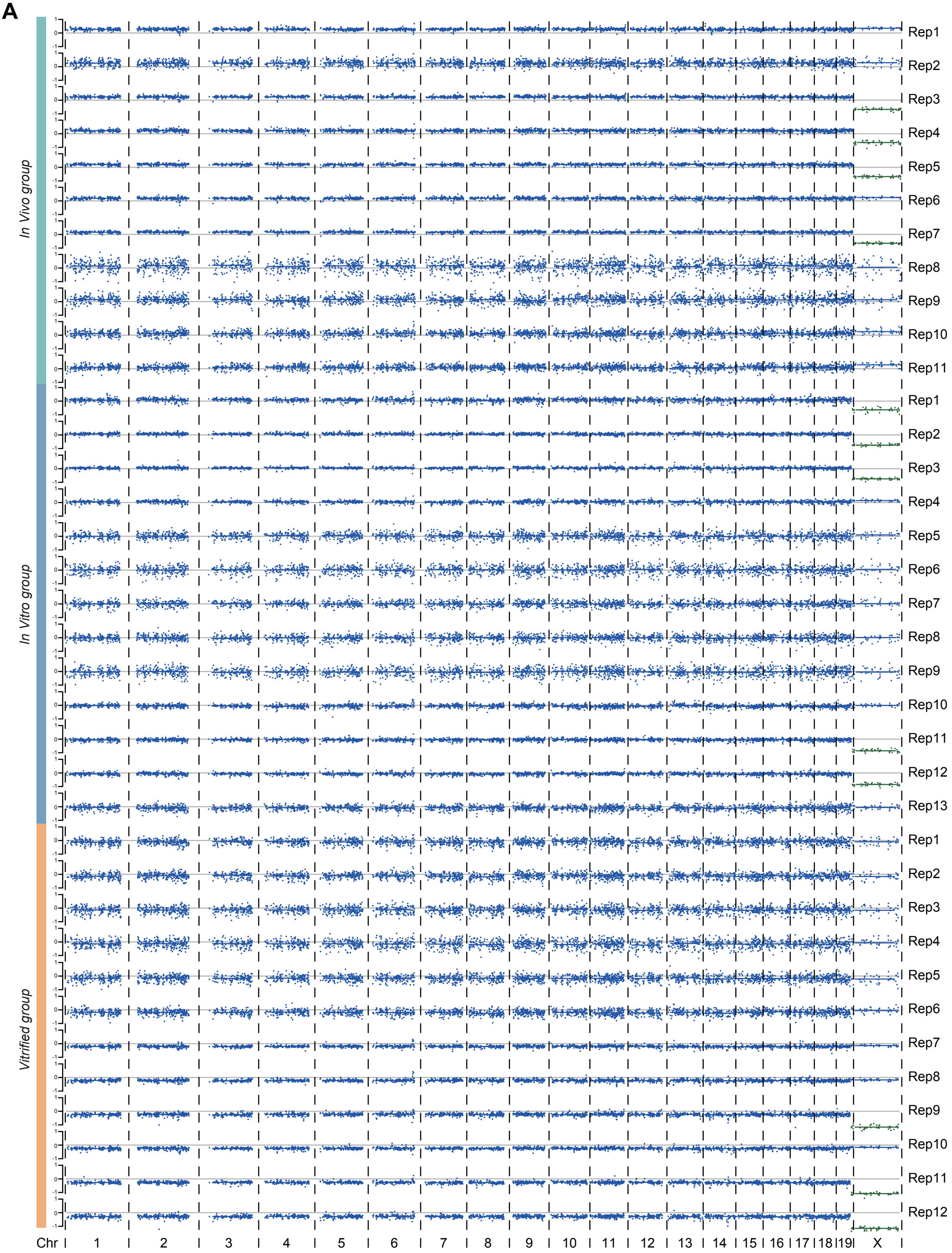
A
In Vivo group
In Vitro group
Vitrified group
Rep1
Rep2
Rep3
Rep4
Rep5
Rep6
Rep7
Rep8
Rep9
Rep10
Rep11
Rep1
Rep2
Rep3
Rep4
Rep5
Rep6
Rep7
Rep8
Rep9
Rep10
Rep11
Rep12
Rep13
Rep1
Rep2
Rep3
Rep4
Rep5
Rep6
Rep7
Rep8
Rep9
Rep10
Rep11
Rep12
Chr 1 2 3 4 5 6 7 8 9 10 11 12 13 14 15 16 17 18 19 X

Figure S4

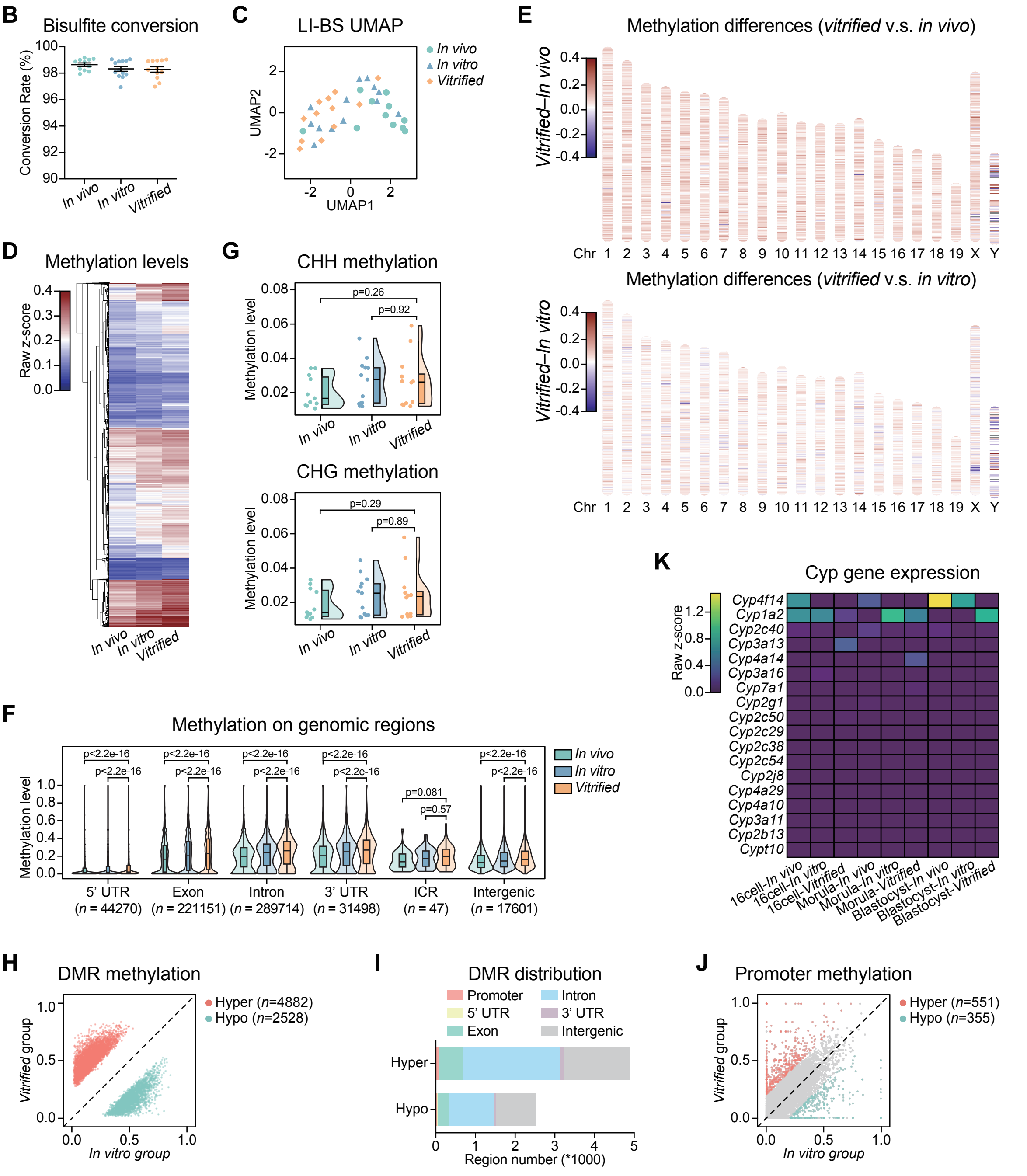
B
Bisulfite conversion
Conversion Rate (%)
100
98
96
94
92
90
In vivo
In vitro
Vitrified
C
LI-BS UMAP
UMAP2
UMAP1
In vivo
In vitro
Vitrified
E
Methylation differences (vitrified v.s. in vivo)
Vitrified–In vivo
Chr 1 2 3 4 5 6 7 8 9 10 11 12 13 14 15 16 17 18 19 X Y
Methylation differences (vitrified v.s. in vitro)
Vitrified–In vitro
D
Methylation levels
Raw z-score
G
CHH methylation
Methylation level
p=0.26
p=0.92
CHG methylation
p=0.29
p=0.89
K
Cyp gene expression
Raw z-score
Cyp4f14
Cyp1a2
Cyp2c40
Cyp3a13
Cyp4a14
Cyp3a16
Cyp7a1
Cyp2g1
Cyp2c50
Cyp2c29
Cyp2c38
Cyp2c54
Cyp2j8
Cyp4a29
Cyp4a10
Cyp3a11
Cyp2b13
Cypt10
16cell-In vivo
16cell-In vitro
16cell-Vitrified
Morula-In vivo
Morula-In vitro
Morula-Vitrified
Blastocyst-In vivo
Blastocyst-In vitro
Blastocyst-Vitrified
F
Methylation on genomic regions
Methylation level
p<2.2e-16
p=0.081
p=0.57
5' UTR (n = 44270)
Exon (n = 221151)
Intron (n = 289714)
3' UTR (n = 31498)
ICR (n = 47)
Intergenic (n = 17601)
H
DMR methylation
Hyper (n=4882)
Hypo (n=2528)
Vitrified group
In vitro group
I
DMR distribution
Promoter
Intron
5' UTR
3' UTR
Exon
Intergenic
Hyper
Hypo
Region number (*1000)
J
Promoter methylation
Hyper (n=551)
Hypo (n=355)
Vitrified group
In vitro group

Figure S4. Analyses of mouse blastocyst LI-BS data (corresponds to Figure 4).
(A) Dot plots showing the CNVs across each chromosome in all blastocyst LI-BS replicates. The entire genome was segmented into 500-kb bins, and the copy number of each bin was calculated. In the scatter plots, each point corresponds to an individual bin. The short horizontal lines represent the average copy number of the corresponding chromosome. The y-axis represents the log2 ratio of the copy number of each bin to the average copy number within the entire chromosome. Green dots indicate the loss of copy numbers, while blue dots indicate the normal diploid copy number. Chr, chromosome. Rep, replicate.
(B) Scatter plots with mean and SEM showing the bisulfite conversion rates in all blastocyst LI-BS replicates (n = 11 for *In vivo*, n = 13 for *In vitro* and n = 12 for *vitrified*).
(C) UMAP visualization of the blastocyst LI-BS dataset showing the two-dimensional embedding and clustering of all replicates. The entire genome was segmented into 100-kb bins, and the average CpG methylation level of each bin was calculated. Bins with at least three CpG sites were retained. The data matrix containing methylation levels of all bins for each replicate was used in UMAP analysis.
(D) Heatmap depicting displaying the average CpG methylation levels of genome-wide 100-kb bins in the blastocyst LI-BS samples. Each row represents a bin, with hierarchical clustering performed on the bins.
(E) Chromosomal maps illustrating the distribution of CpG methylation differences between *vitrified group* and control groups across the genome at the blastocyst stage. The genome was segmented into 100-kb bins, and the methylation difference of each bin was calculated as the CpG methylation level of *vitrified group* minus that of *in vivo group* (upper panel) or *in vitro group* (lower panel). Each vertical column corresponds to a chromosome, and each horizontal strip represents an individual bin with the color indicating the methylation difference (red indicates hypermethylation and blue indicates hypomethylation in *vitrified group*). Chr, chromosome.
(F) Violin plots with embedded box plots showing the methylation levels across the indicated genomic elements at the blastocyst stage. The average CpG methylation level of each region in each group was used for plotting. ICR, imprinting control region.
(G) Raincloud plots showing the methylation levels on genome-wide CHH sites (upper panel) and CHG sites (lower panel) at the blastocyst stage. Each point corresponds to an individual replicate (n = 11 for *In vivo*, n = 13 for *In vitro* and n = 12 for *vitrified*).
(H, J) Scatter plots showing the methylation levels of all DMRs identified in *vitrified group* (H) and all promoter regions (J) at the blastocyst stage. Each point represents the methylation level of an individual region, with the x-axis and y-axis represents the level in *vitrified group* and *in vitro group*, respectively. The labeled points represent regions with absolute methylation differences between *vitrified group* and *in vitro group* greater than 0.2. These regions and promoters are listed in Table S3. Hyper, hypermethylated. Hypo, hypomethylated.
(I) Stacked bar chart displaying the distribution of DMRs identified in *vitrified group* at the blastocyst stage across indicated genomic elements.
(K) Heatmap depicting the expression levels of hypermethylated CYP genes in embryos at each stage. The promoter regions of these genes were hypermethylated in *vitrified group* compared to *in vitro group* at the blastocyst stage.

Figure S5

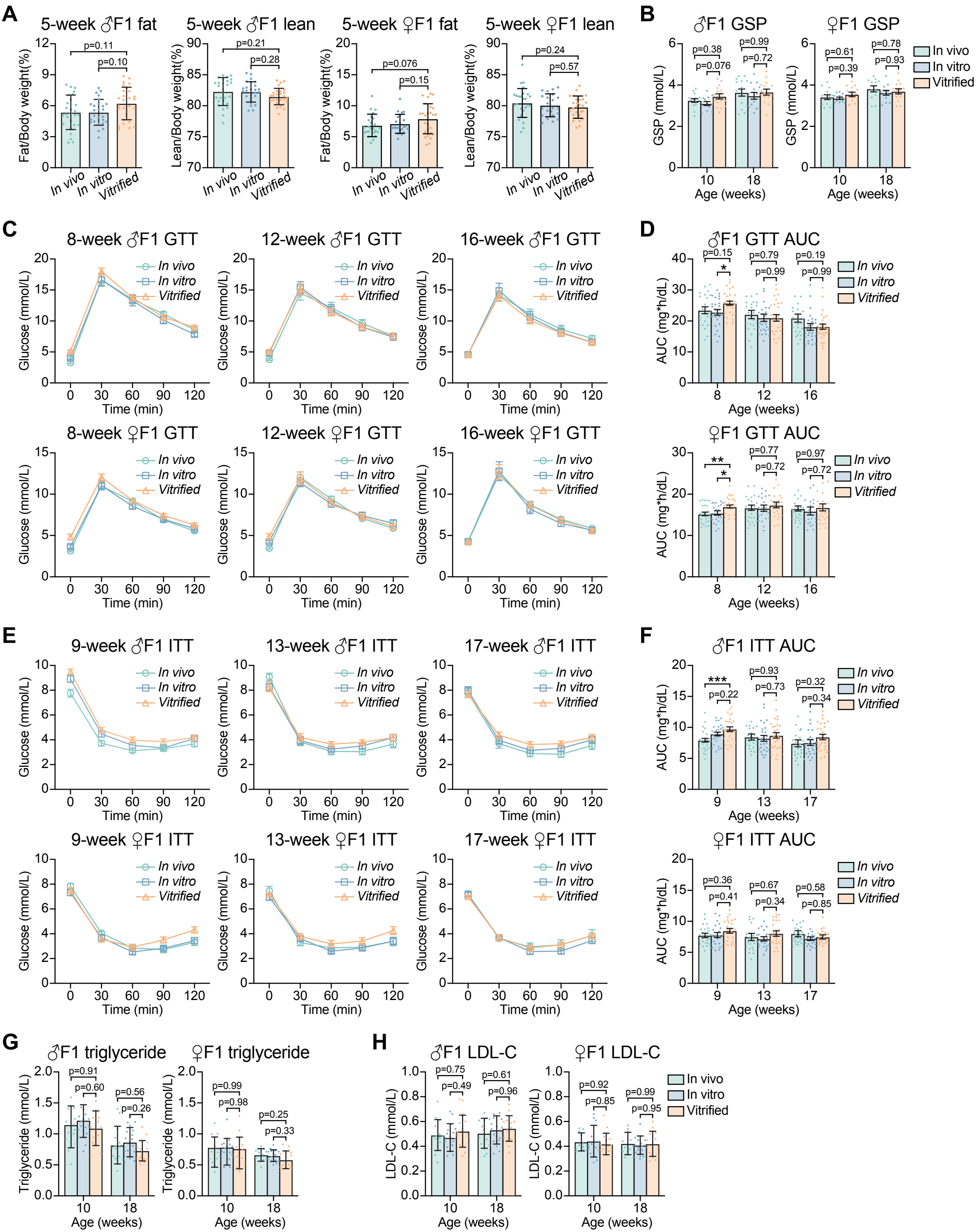
A
5-week ♂F1 fat
Fat/Body weight(%)
p=0.11
p=0.10
5-week ♂F1 lean
Lean/Body weight(%)
p=0.21
p=0.28
5-week ♀F1 fat
Fat/Body weight(%)
p=0.076
p=0.15
5-week ♀F1 lean
Lean/Body weight(%)
p=0.24
p=0.57
In vivo
In vitro
Vitrified
B
♂F1 GSP
GSP (mmol/L)
p=0.38
p=0.076
p=0.99
p=0.72
♀F1 GSP
p=0.61
p=0.39
p=0.78
p=0.93
Age (weeks)
In vivo
In vitro
Vitrified
C
8-week ♂F1 GTT
12-week ♂F1 GTT
16-week ♂F1 GTT
8-week ♀F1 GTT
12-week ♀F1 GTT
16-week ♀F1 GTT
Glucose (mmol/L)
Time (min)
In vivo
In vitro
Vitrified
D
♂F1 GTT AUC
AUC (mg*h/dL)
p=0.15
*
p=0.79
p=0.99
p=0.19
p=0.99
♀F1 GTT AUC
**
*
p=0.77
p=0.72
p=0.97
p=0.72
Age (weeks)
E
9-week ♂F1 ITT
13-week ♂F1 ITT
17-week ♂F1 ITT
9-week ♀F1 ITT
13-week ♀F1 ITT
17-week ♀F1 ITT
Glucose (mmol/L)
Time (min)
F
♂F1 ITT AUC
AUC (mg*h/dL)
***
p=0.22
p=0.93
p=0.73
p=0.32
p=0.34
♀F1 ITT AUC
p=0.36
p=0.41
p=0.67
p=0.34
p=0.58
p=0.85
Age (weeks)
G
♂F1 triglyceride
Triglyceride (mmol/L)
p=0.91
p=0.60
p=0.56
p=0.26
♀F1 triglyceride
p=0.99
p=0.98
p=0.25
p=0.33
Age (weeks)
H
♂F1 LDL-C
LDL-C (mmol/L)
p=0.75
p=0.49
p=0.61
p=0.96
♀F1 LDL-C
p=0.92
p=0.85
p=0.99
p=0.95
Age (weeks)
In vivo
In vitro
Vitrified

Figure S5. Measurement of metabolic parameters in mouse offspring derived from embryos (corresponds to Figure 5).

(A) Bar charts displaying the proportion of lean mass and fat mass relative to body weight in 5-week-old offspring (*n* = 27 males and 24 females for *in vivo*, *n* = 26 males and 22 females for *in vitro*, *n* = 27 males and 26 females for *vitrified*).

(B) Bar charts displaying the serum GSP levels of offspring after 4-h fasting at the indicated weeks of age (*n* = 15 males and 12 females for *in vivo*, *n* = 13 males and 13 females for *in vitro*, *n* = 16 males and 14 females for *vitrified*).

(C, E) Line charts displaying the blood glucose levels of offspring during the GTT (C) and ITT (E) at the indicated weeks of age. In each test, *n* = 23 males and 26 females for *in vivo*, *n* = 25 males and 21 females for *in vitro*, *n* = 27 males and 24 females for *vitrified*.

(D, F) Bar charts displaying the AUC of the glucose levels measured during the GTT (D) and ITT (F) at the indicated weeks of age. The male (upper panels) and female (lower panels) offspring were measured separately.

(G, H) Bar charts displaying the serum triglyceride levels (G) and serum LDL-C levels (H) of offspring after 4-h fasting at the indicated weeks of age. In each measurement, *n* = 14 males and 12 females for *in vivo*, *n* = 16 males and 15 females for *in vitro*, *n* = 14 males and 13 females for *vitrified*.

For the plots in (A, B, D and F–H), each point represents the value of an individual offspring.

Figure S6

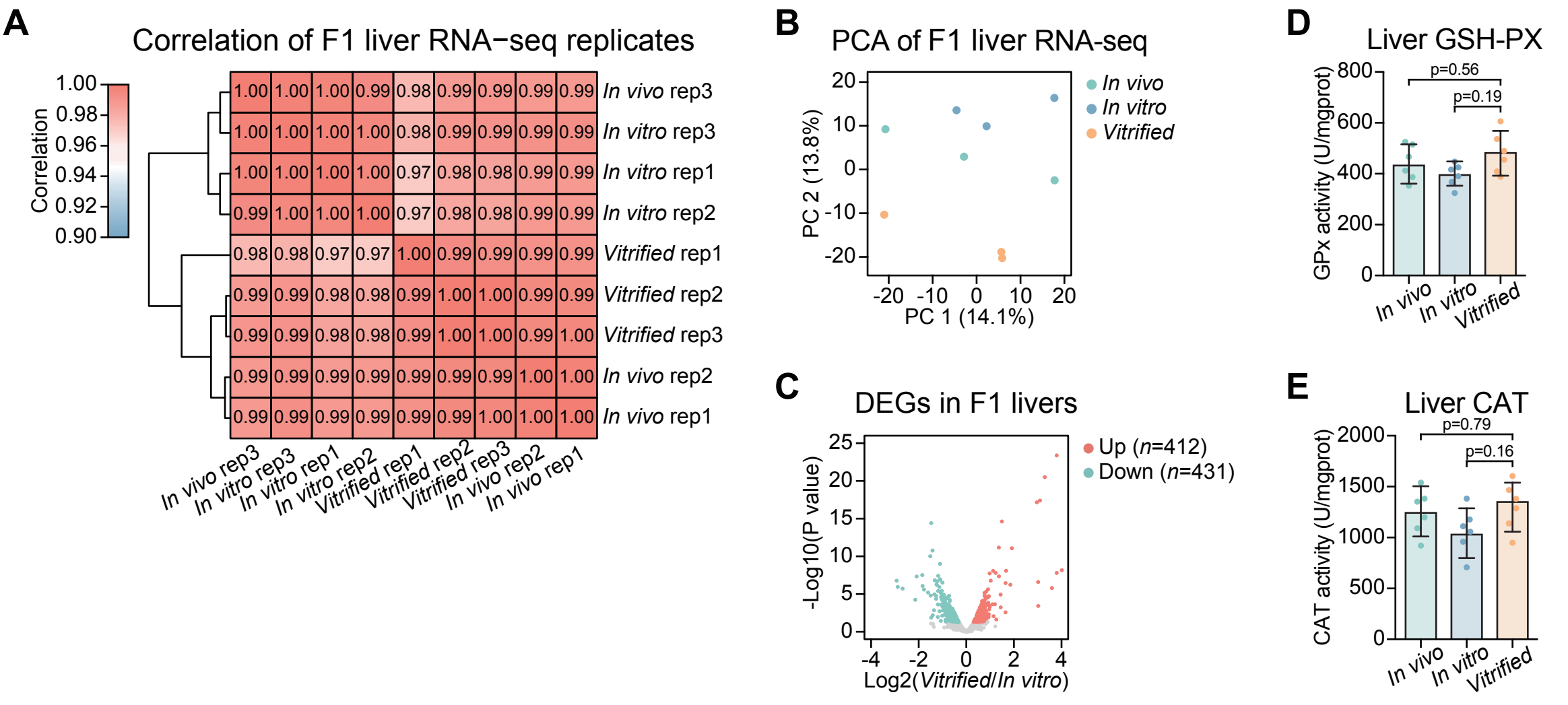

Figure S6. Analyses of liver mRNA-seq data and measurement of metabolites in livers for offspring derived from embryos (corresponds to Figure 6).

(A) Heatmap depicting the hierarchical clustering of all liver mRNA-seq replicates from 10-week-old female offspring, based on their pairwise Pearson's correlation coefficients. The data matrix containing TPM of all genes (*n* = 24421) was used for this calculation. Rep, replicate.

(B) PCA of all liver mRNA-seq replicates showing top two principal components that contribute to variance. PCA was performed with the expression levels of all genes (*n* = 24421).

(C) Volcano plot showing the results of DEG calling for liver mRNA-seq samples from 10-week-old female offspring. The x-axis represents the fold change of all genes in the *vitrified group* compared to the *in vitro group*, and the y-axis represents the P values provided by edgeR. The labeled points represent confident DEGs of the *vitrified group*.

(D, E) Bar charts displaying the levels of GSH-PX (D) and CAT (E) in the liver tissues of 10-week-old female offspring. Each point represents the value of an individual replicate (*n* = 6 for each group in each measurement).

Figure S7

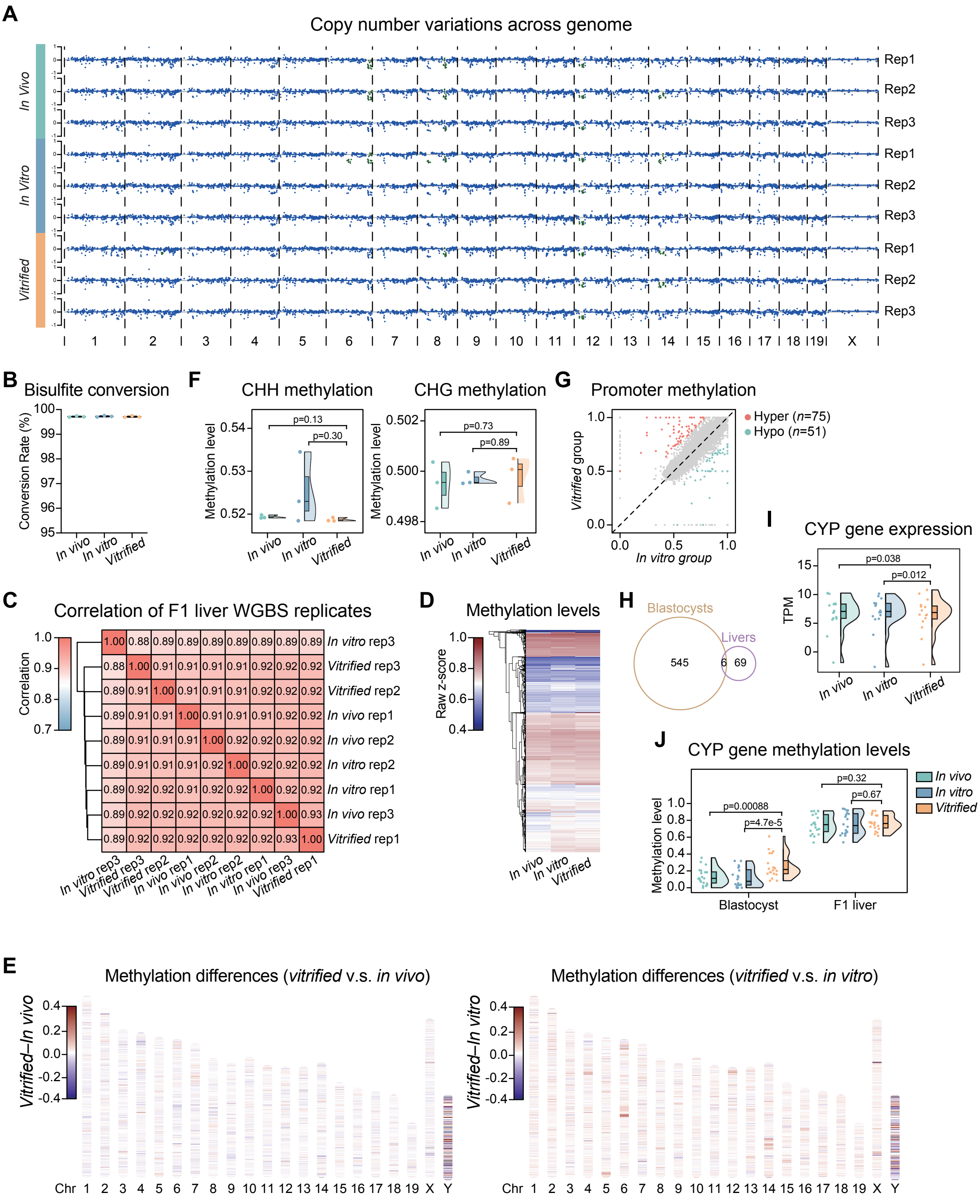

A
Copy number variations across genome
In Vivo
In Vitro
Vitrified
Rep1
Rep2
Rep3
1 2 3 4 5 6 7 8 9 10 11 12 13 14 15 16 17 18 19 X
B
Bisulfite conversion
Conversion Rate (%)
In vivo
In vitro
Vitrified
F
CHH methylation
CHG methylation
Methylation level
p=0.13
p=0.30
p=0.73
p=0.89
G
Promoter methylation
Vitrified group
In vitro group
Hyper (n=75)
Hypo (n=51)
C
Correlation of F1 liver WGBS replicates
Correlation
In vitro rep3
Vitrified rep3
Vitrified rep2
In vivo rep1
In vivo rep2
In vitro rep2
In vitro rep1
In vivo rep3
Vitrified rep1
D
Methylation levels
Raw z-score
H
Blastocysts
Livers
545
6
69
I
CYP gene expression
TPM
p=0.038
p=0.012
J
CYP gene methylation levels
Methylation level
p=0.00088
p=4.7e-5
p=0.32
p=0.67
Blastocyst
F1 liver
E
Methylation differences (vitrified v.s. in vivo)
Methylation differences (vitrified v.s. in vitro)
Vitrified–In vivo
Vitrified–In vitro
Chr 1 2 3 4 5 6 7 8 9 10 11 12 13 14 15 16 17 18 19 X Y

Figure S7. Analyses of liver WGBS data for offspring derived from embryos (corresponds to Figure 7).
(A) Dot plots showing the CNVs across each chromosome in all liver WGBS replicates from 10-week-old female offspring.
(B) Scatter plots with mean and SEM showing the bisulfite conversion rates in all liver WGBS replicates from 10-week-old female offspring (n = 3 for each group).
(C) Heatmap depicting the hierarchical clustering of all liver WGBS replicates from 10-week-old female offspring, based on their pairwise Pearson's correlation coefficients. The data matrix containing methylation levels of all gene promoters (n = 24112) was used for this calculation. Rep, replicate.
(D) Heatmap depicting displaying the average CpG methylation levels of genome-wide 100-kb bins in the liver WGBS samples from 10-week-old female offspring.
(E) Chromosomal maps illustrating the distribution of CpG methylation differences between *vitrified group* and *in vivo group* (left panel) or *vitrified group* and *in vitro group* (right panel) across the genome in the livers of 10-week-old female offspring.
(F) Raincloud plots showing the methylation levels on genome-wide CHH sites (left panel) and CHG sites (right panel) in the livers of 10-week-old female offspring. Each point corresponds to an individual replicate (n = 3 for each group).
(G) Scatter plots showing the methylation levels of all promoter regions in the livers of 10-week-old female offspring.
(H) Venn diagram illustrating the overlap of hypermethylated promoters identified in blastocysts and offspring's livers of *vitrified group* compared to *in vitro group*. These promoters are labeled in red in Figures S4J and S7G.
(I) Raincloud plots showing the expression levels of specific CYP genes (n = 16) in the livers of 10-week-old female offspring.
(J) Raincloud plots showing the methylation levels of promoter regions for specific CYP genes in blastocysts and offspring's livers.
For (I and J), each point in the scatter plots corresponds to an individual Cyp gene, and the gene set is the same as the set used in Fig. 7H. For the plotting in (A, B and D–G), see Fig. S4A, B, D, E, G, J for detailed explanations.
